# Supplementary figures and images for: Heterogeneous Genomic Divergence Landscape in Two Commercially Important European Scallop Species
Source: Genes (Basel). 2022 Dec 21;14(1):14. doi: 10.3390/genes14010014 (PMC9858869; doi:10.3390/genes14010014)

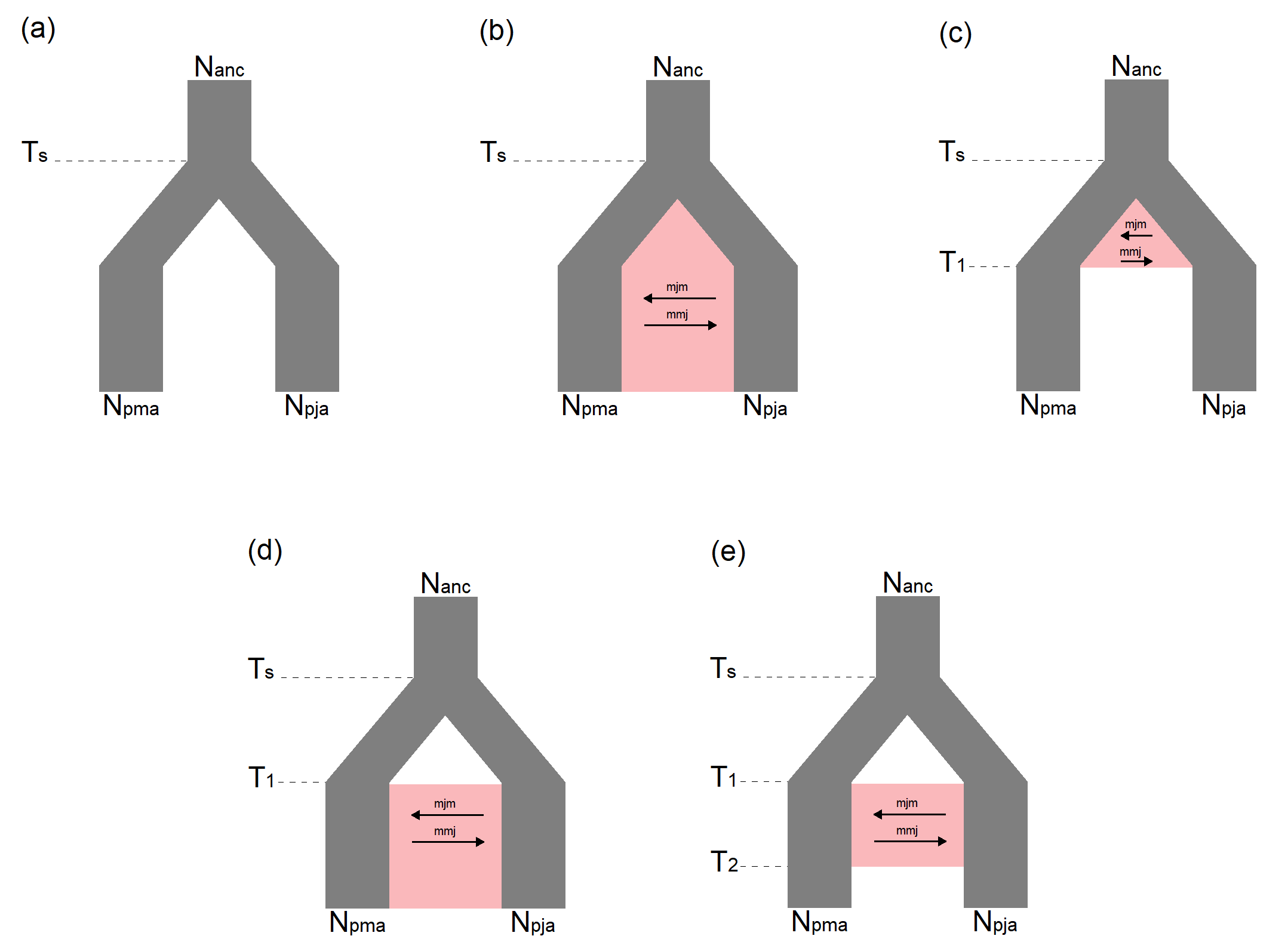

Supplement: Supplementary file 1 [file genes-14-00014-s001.zip › Figure_S1.tif]

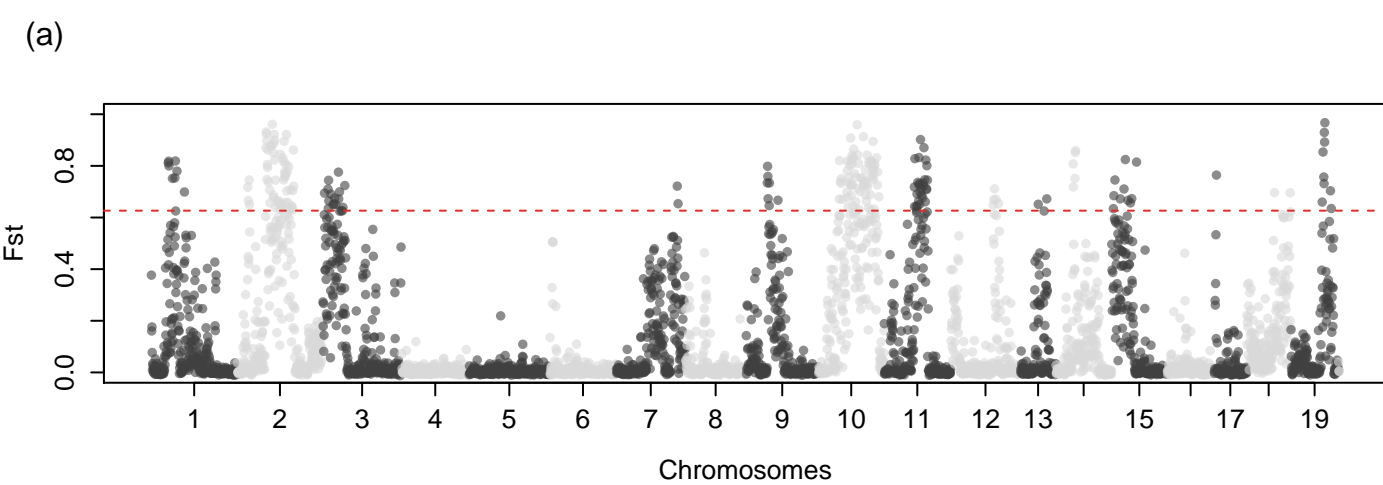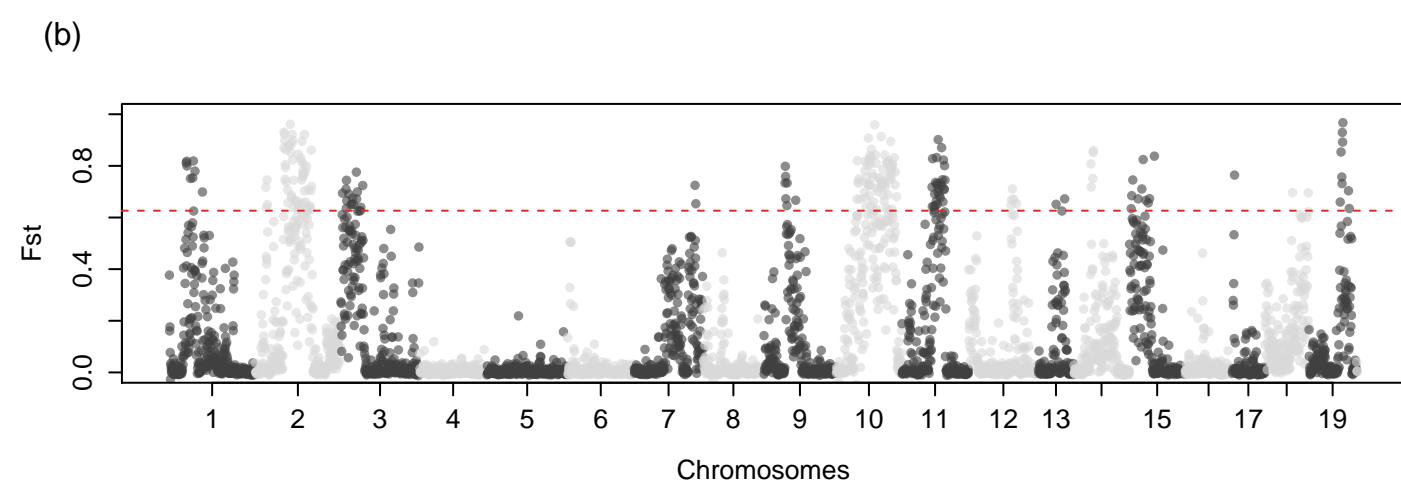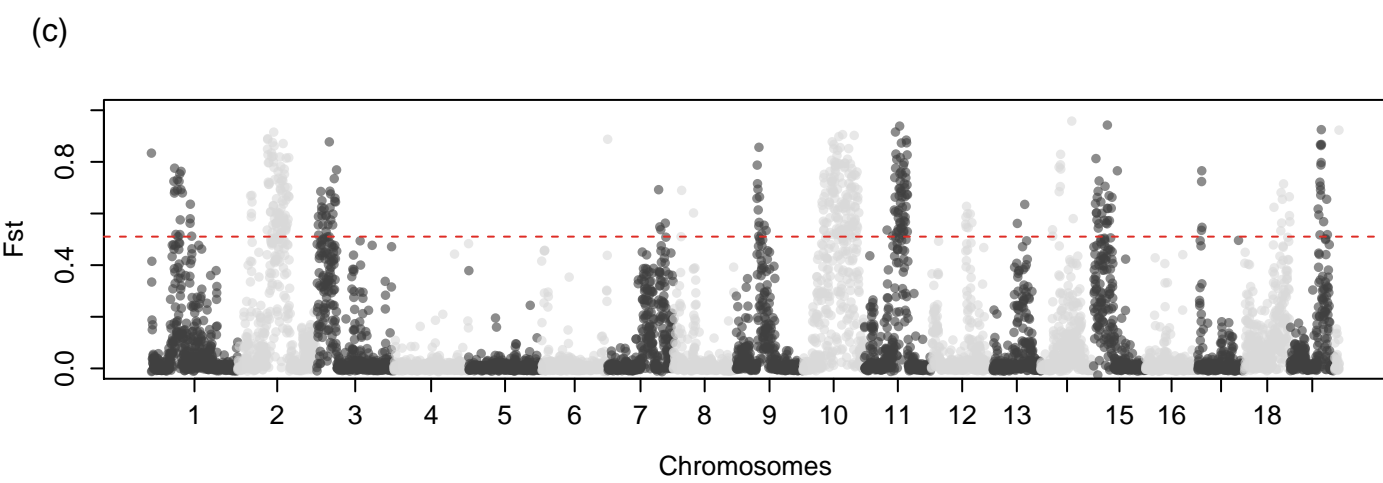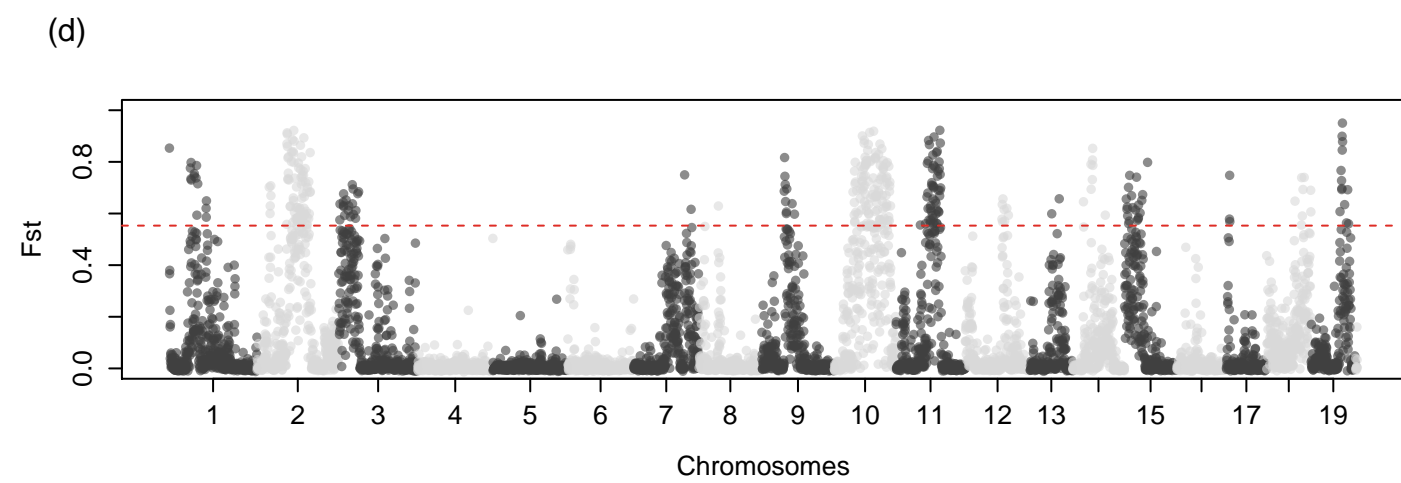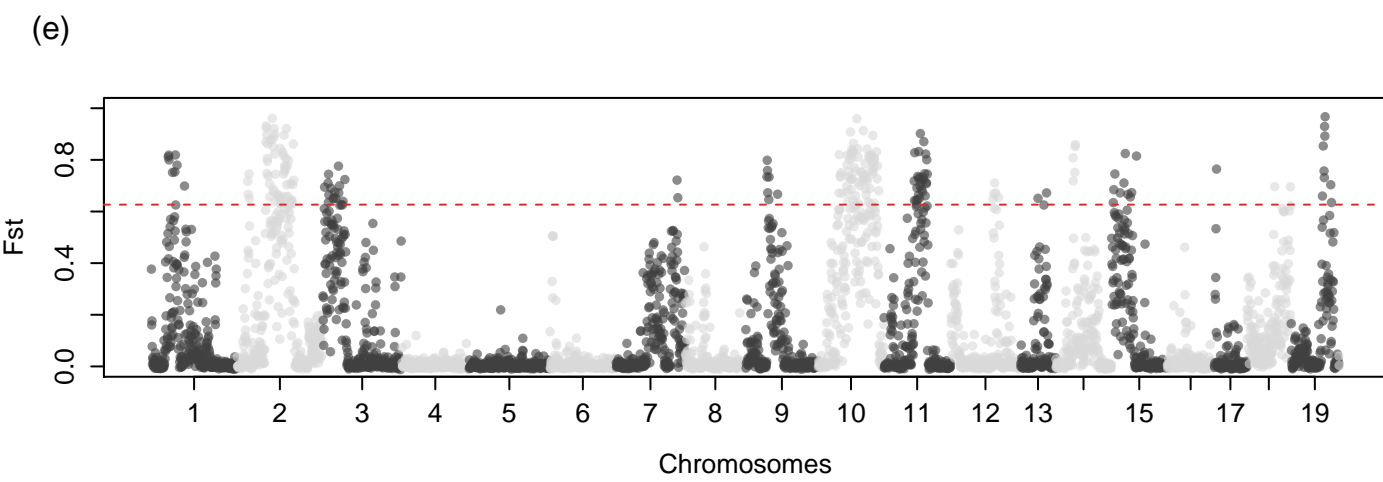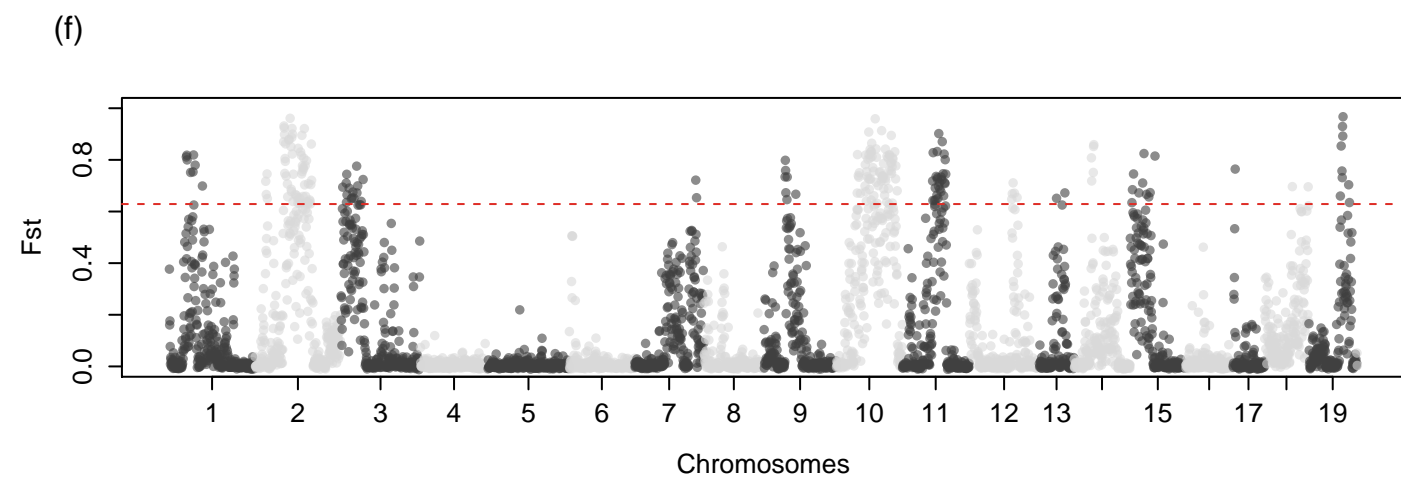

Supplement: Supplementary file 1 [file genes-14-00014-s001.zip › Figure_S2.pdf]

(a) *P. maximus*

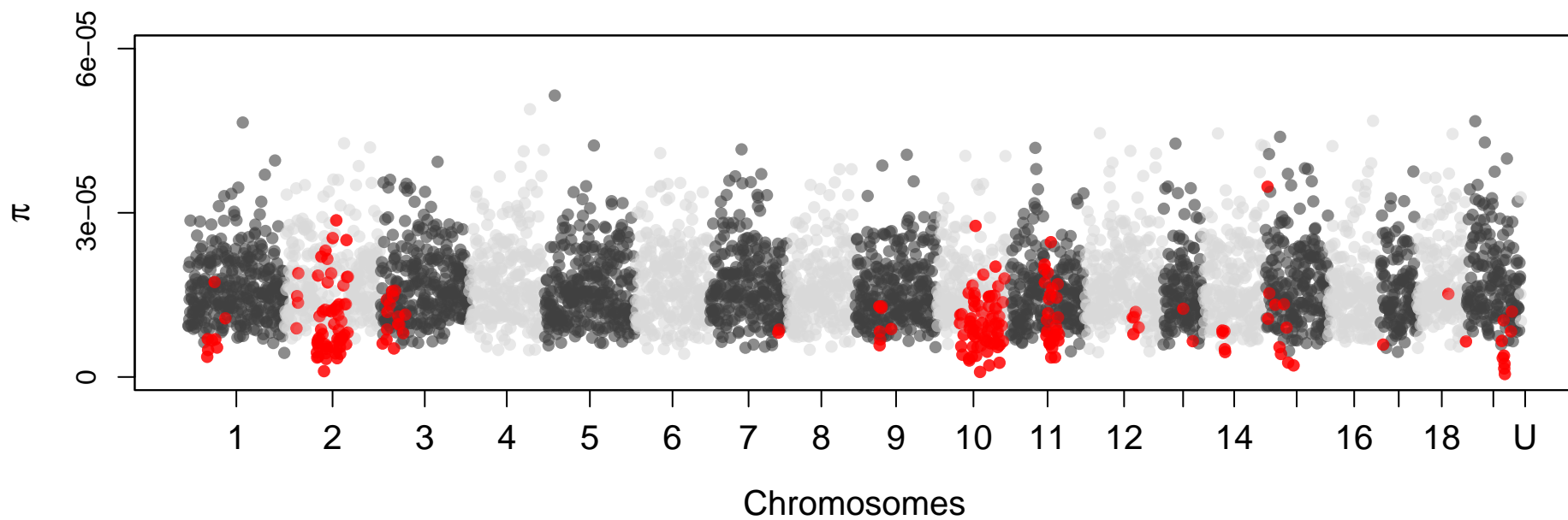

(b) *P. jacobaeus*

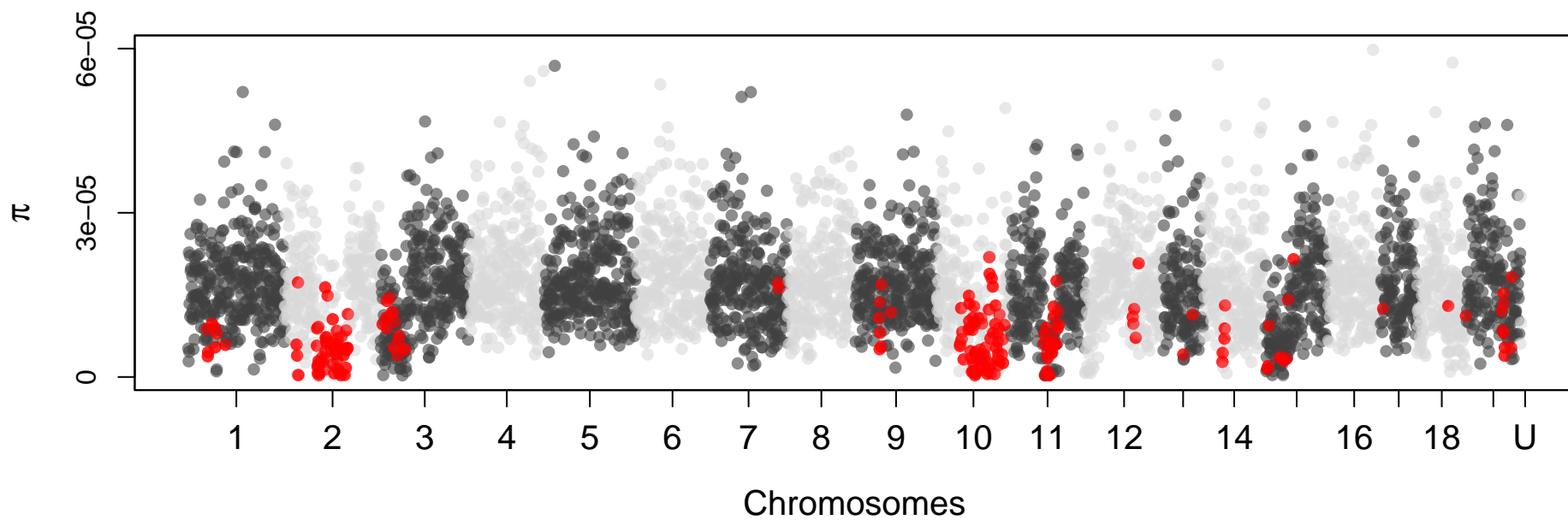

Supplement: Supplementary file 1 [file genes-14-00014-s001.zip › Figure_S3.pdf]

(a)

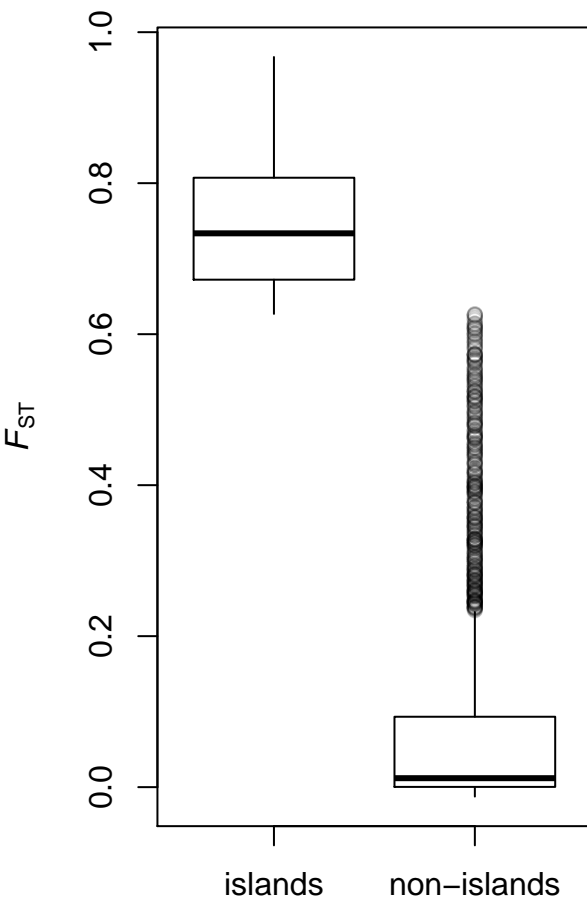

(b)

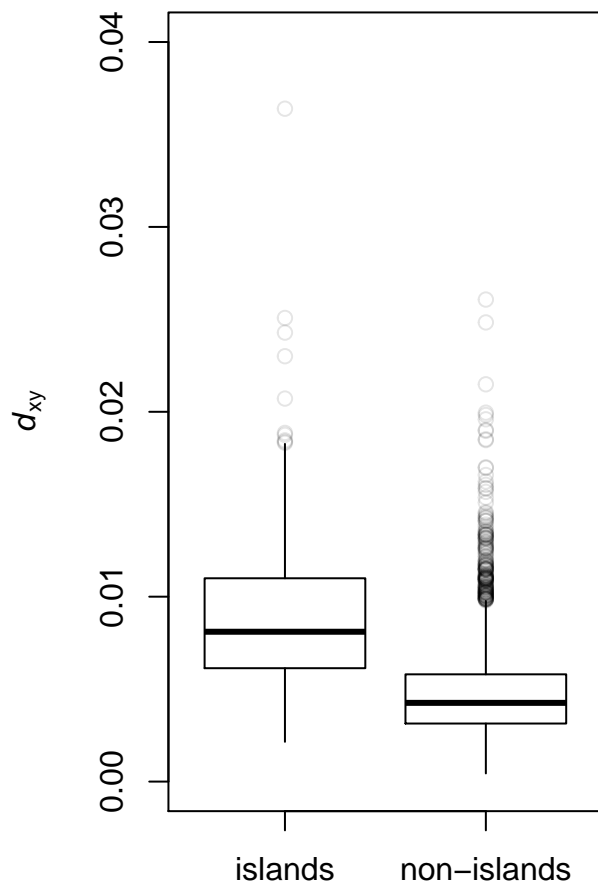

Supplement: Supplementary file 1 [file genes-14-00014-s001.zip › Figure_S4.pdf]

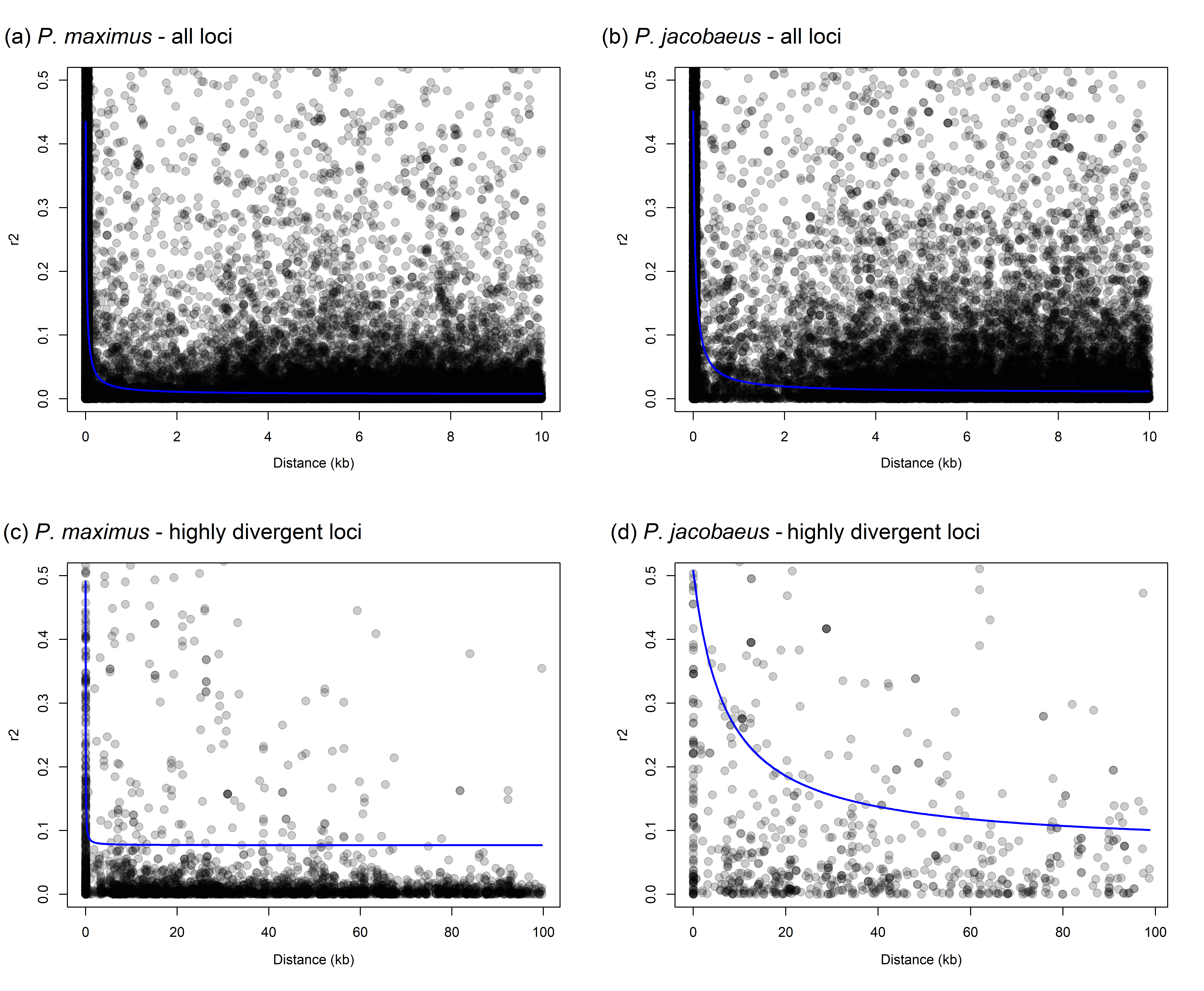

Supplement: Supplementary file 1 [file genes-14-00014-s001.zip › Figure_S5.tiff]
