# Supplementary material for: Heterogeneous Genomic Divergence Landscape in Two Commercially Important European Scallop Species
Source: Genes (Basel). 2022 Dec 21;14(1):14. doi: 10.3390/genes14010014 (PMC9858869; doi:10.3390/genes14010014)
Supplement: Supplementary file 1 [file Figure_S6.pdf › Figure_S6.pdf]

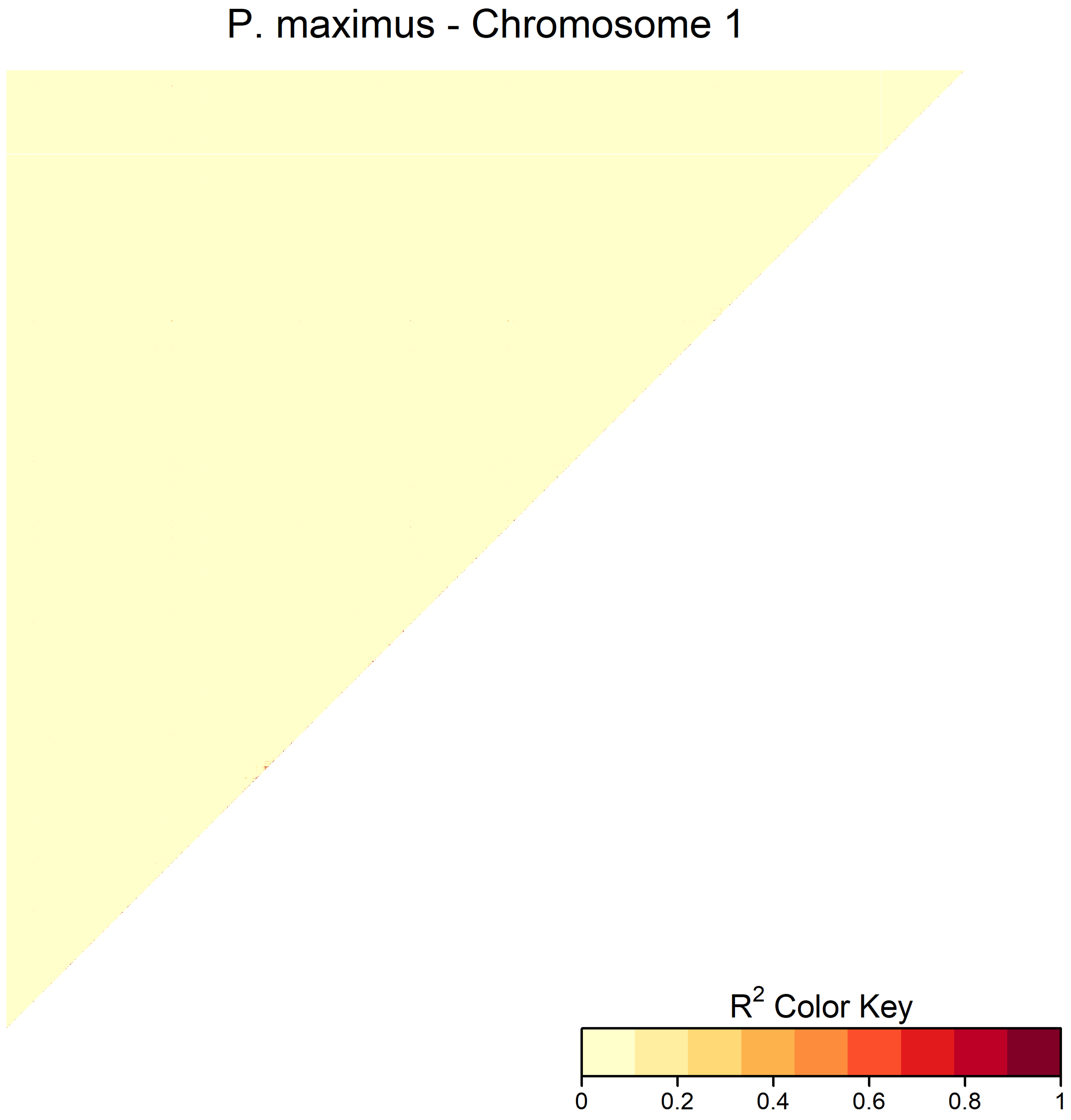
P. maximus - Chromosome 1
R^2 Color Key
0
0.2
0.4
0.6
0.8
1

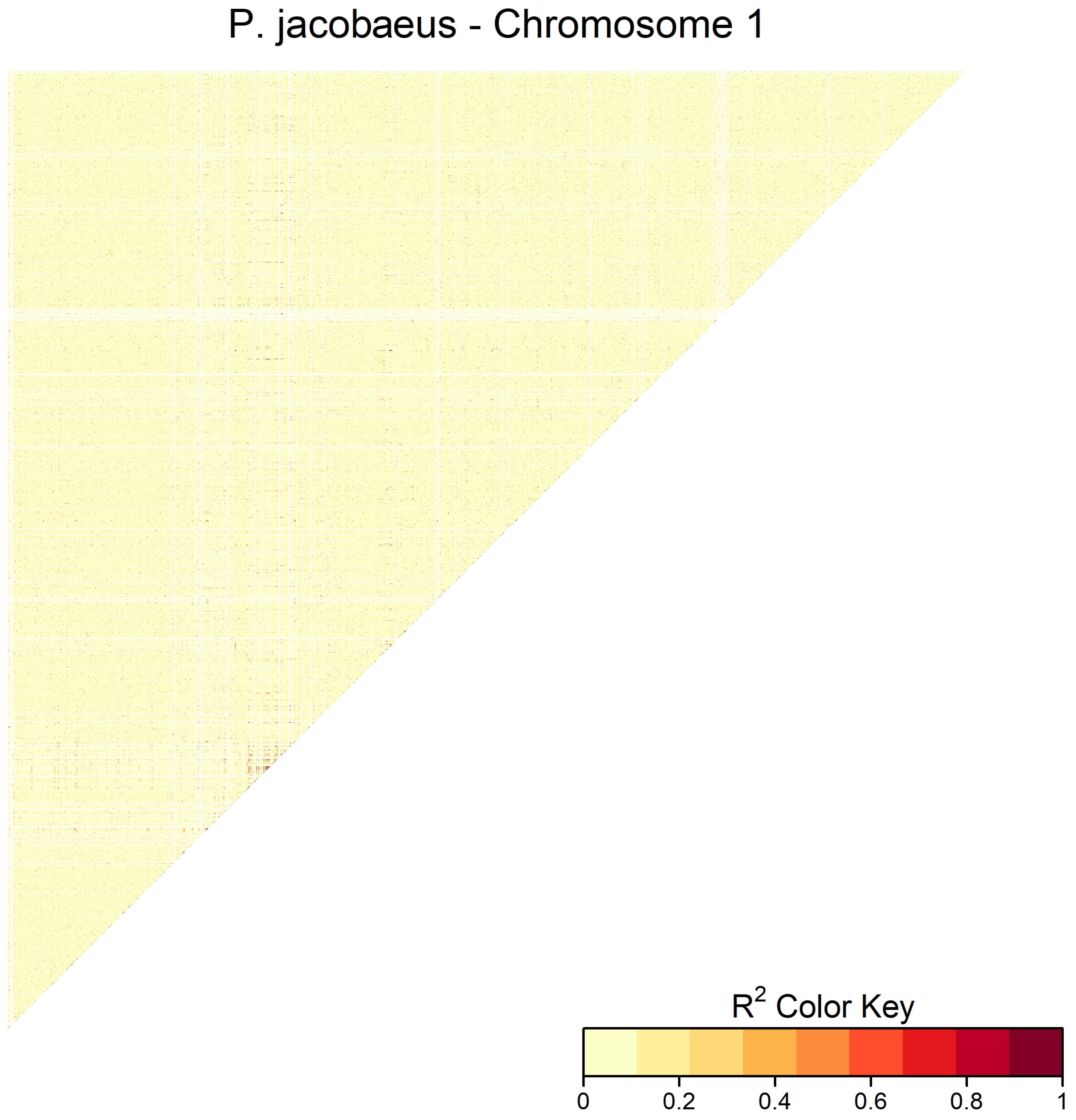
P. jacobaeus - Chromosome 1
R^2 Color Key
0
0.2
0.4
0.6
0.8
1

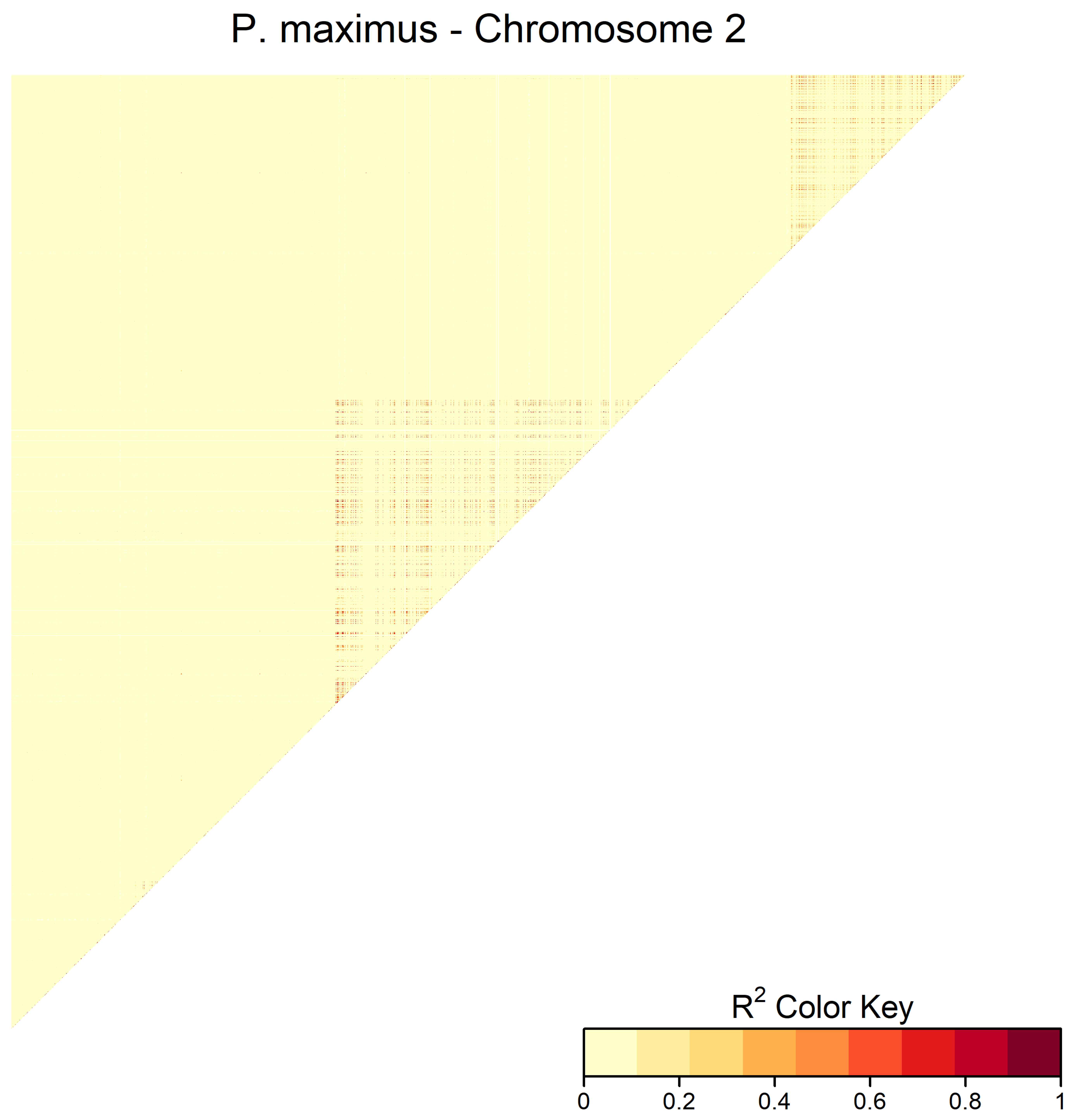
P. maximus - Chromosome 2
R2 Color Key
0
0.2
0.4
0.6
0.8
1

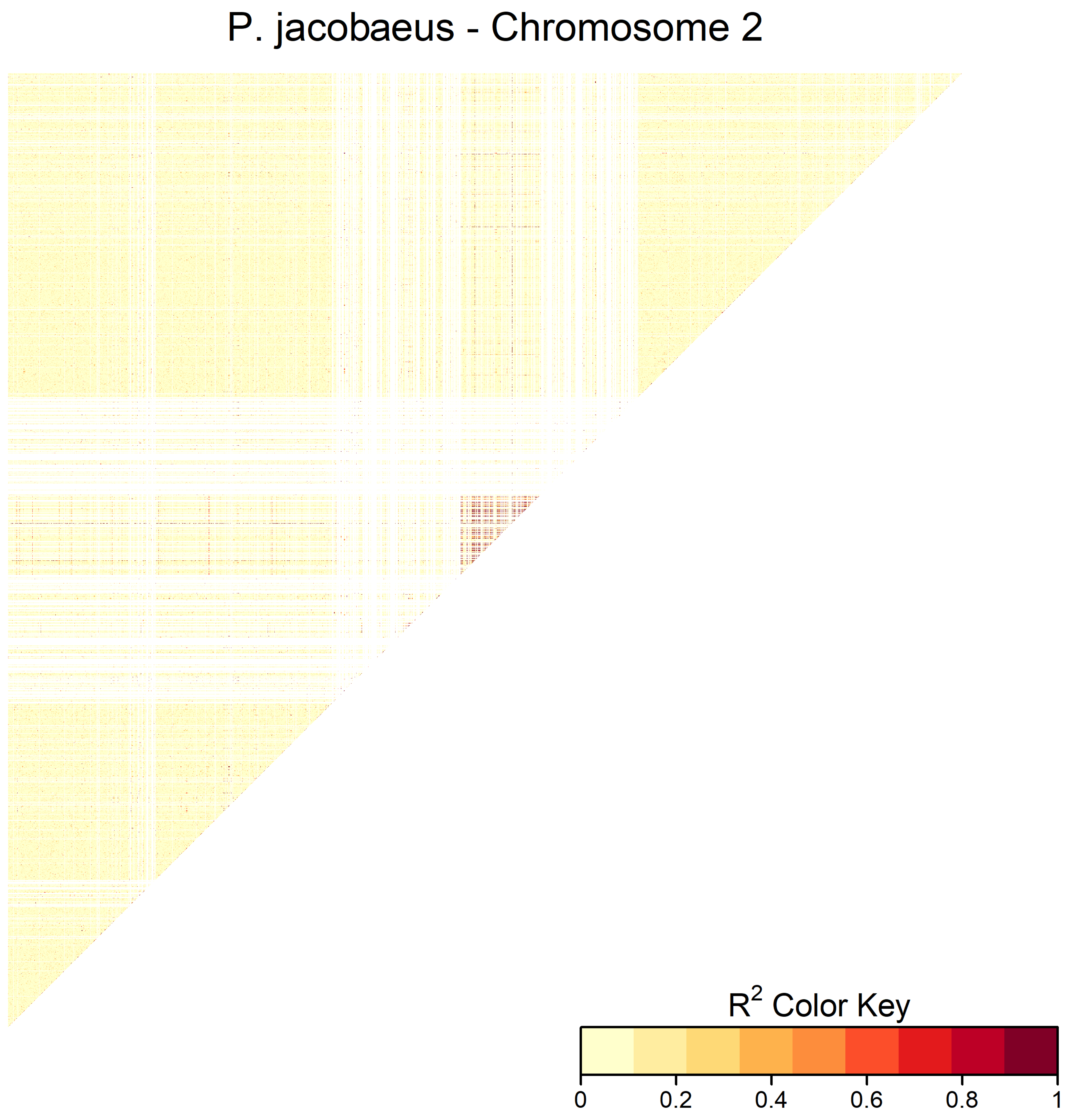
P. jacobaeus - Chromosome 2
R2 Color Key
0
0.2
0.4
0.6
0.8
1

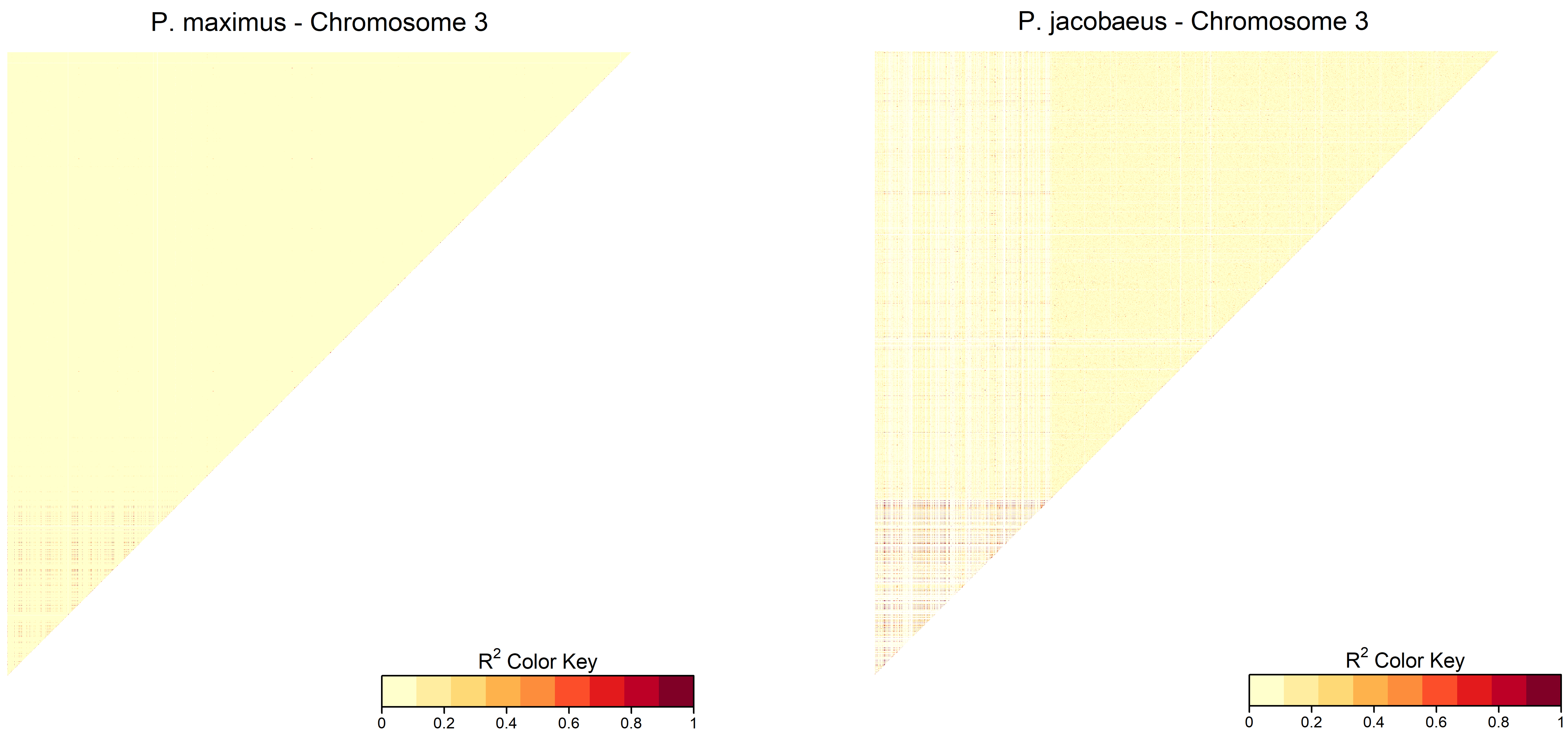
P. maximus - Chromosome 3
R2 Color Key
0
0.2
0.4
0.6
0.8
1
P. jacobaeus - Chromosome 3
R2 Color Key
0
0.2
0.4
0.6
0.8
1

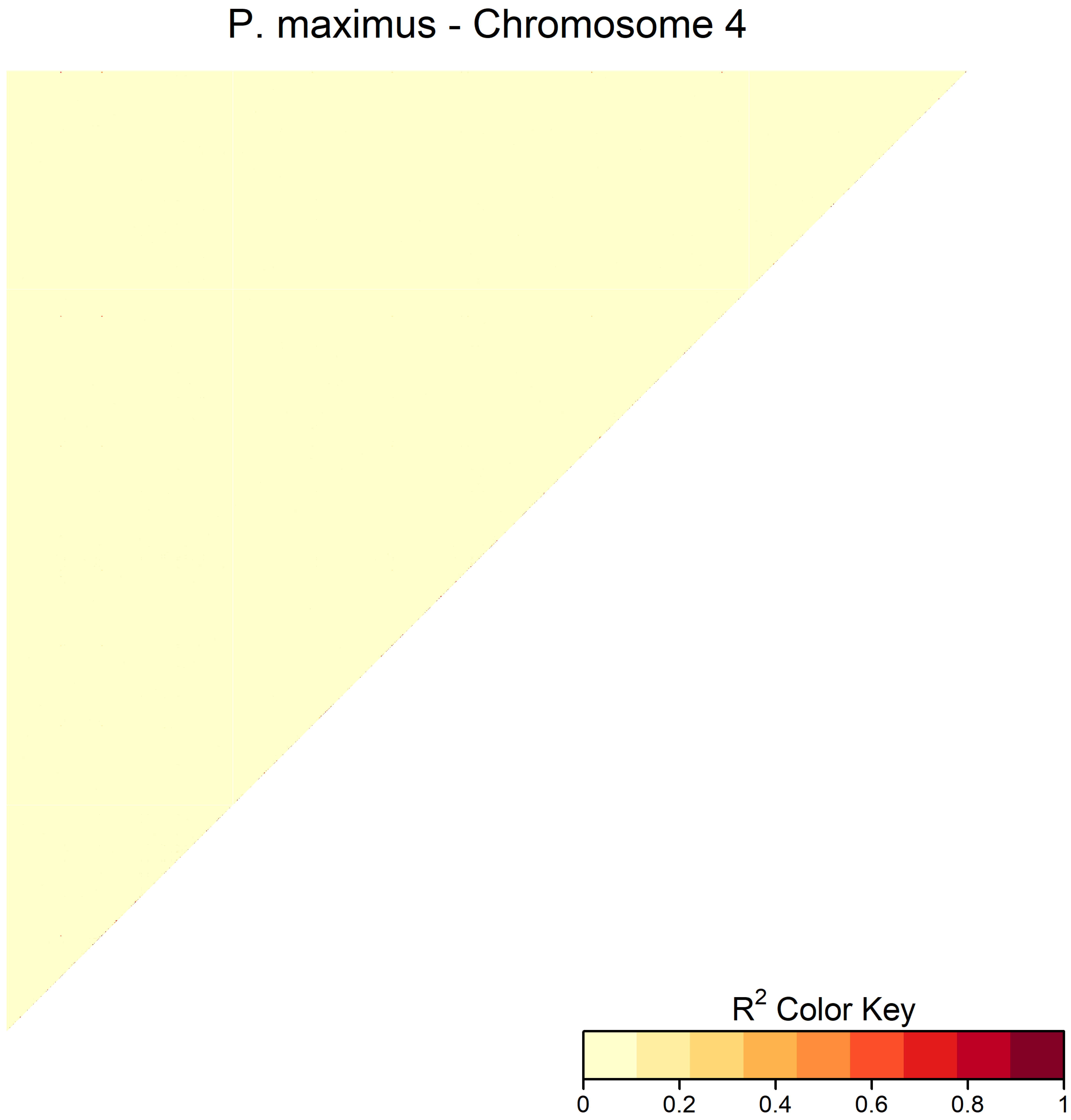
P. maximus - Chromosome 4
R^2 Color Key
0
0.2
0.4
0.6
0.8
1

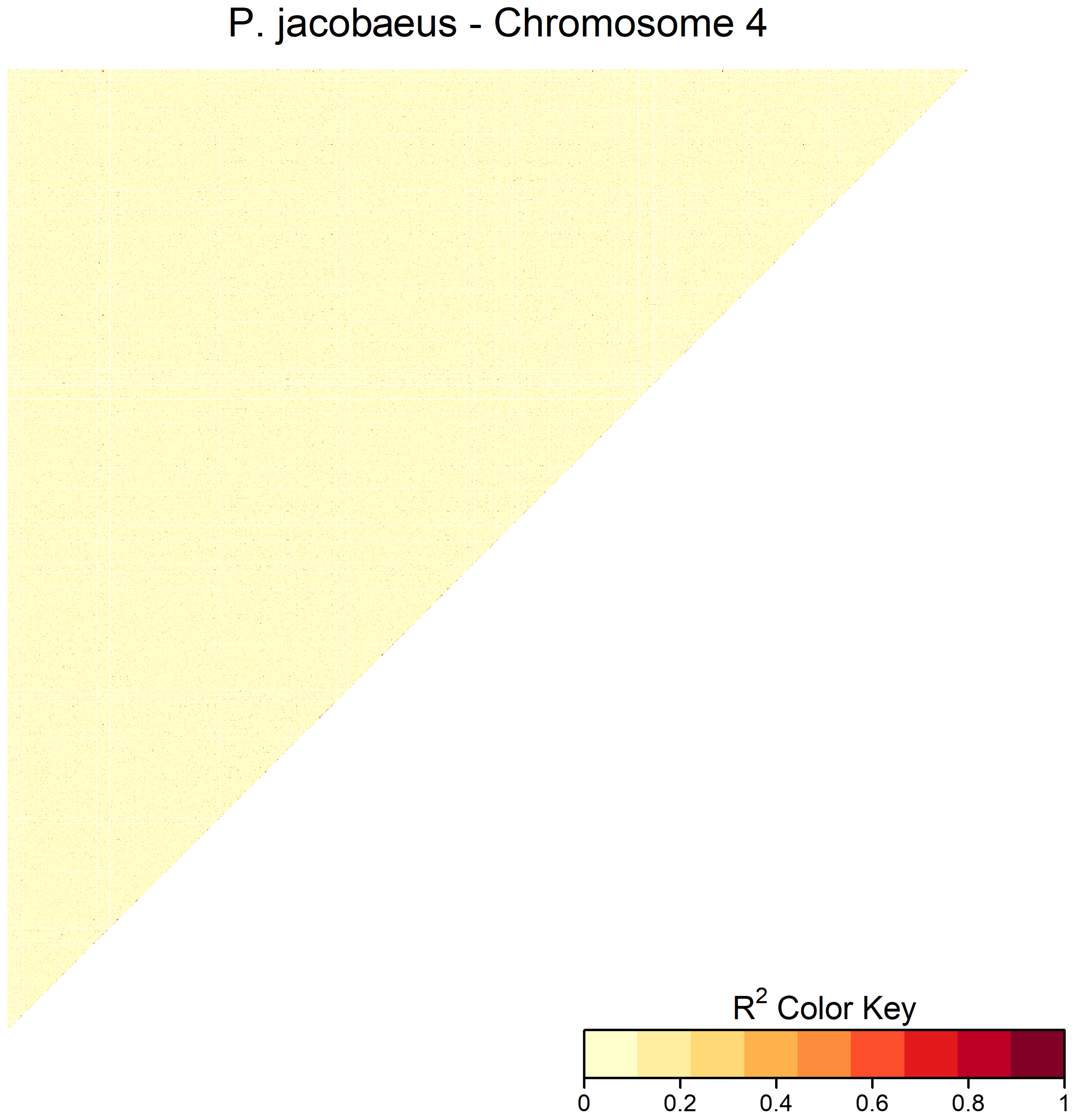
P. jacobaeus - Chromosome 4
R^2 Color Key
0
0.2
0.4
0.6
0.8
1

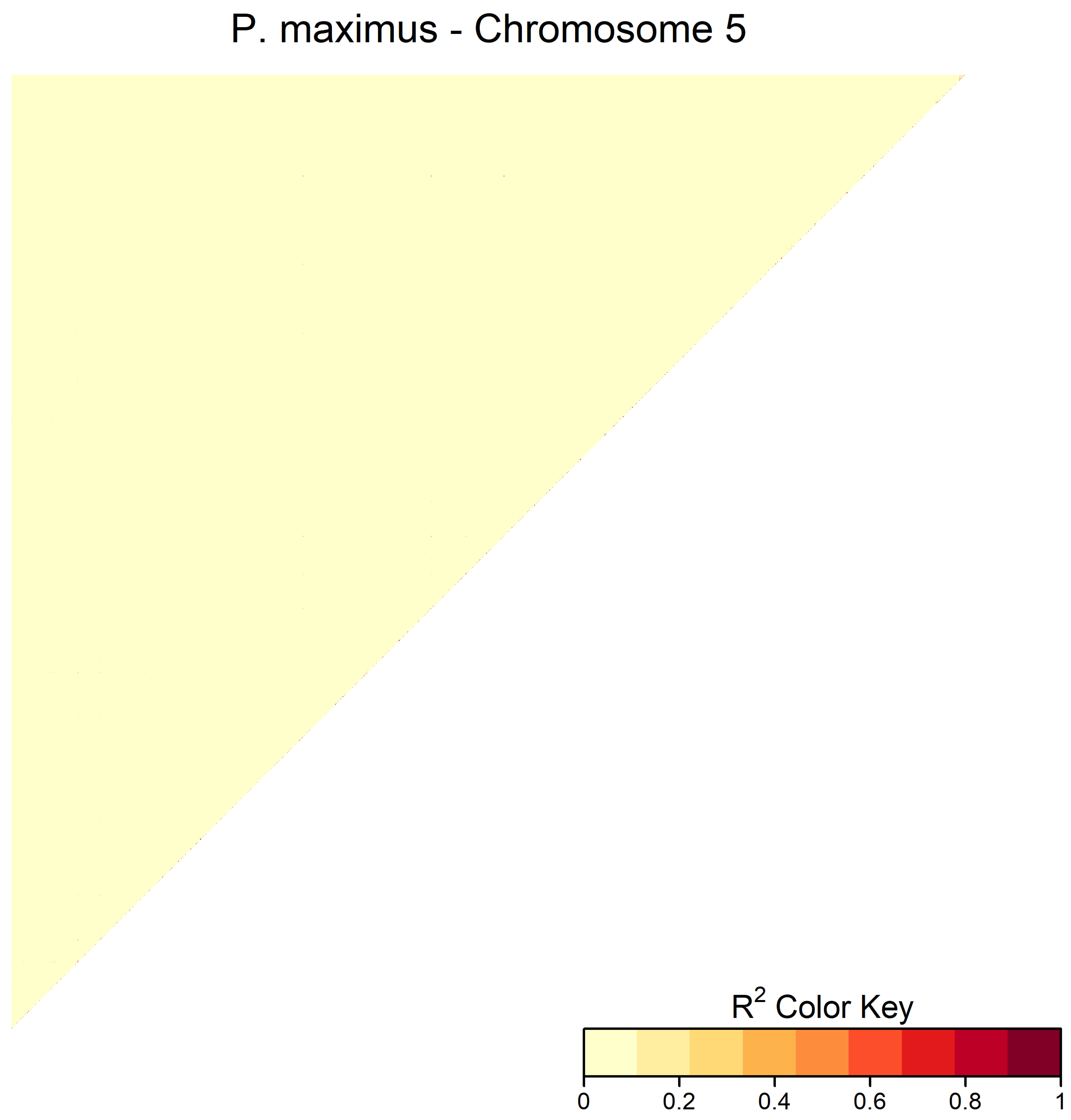
P. maximus - Chromosome 5
R^2 Color Key
0
0.2
0.4
0.6
0.8
1

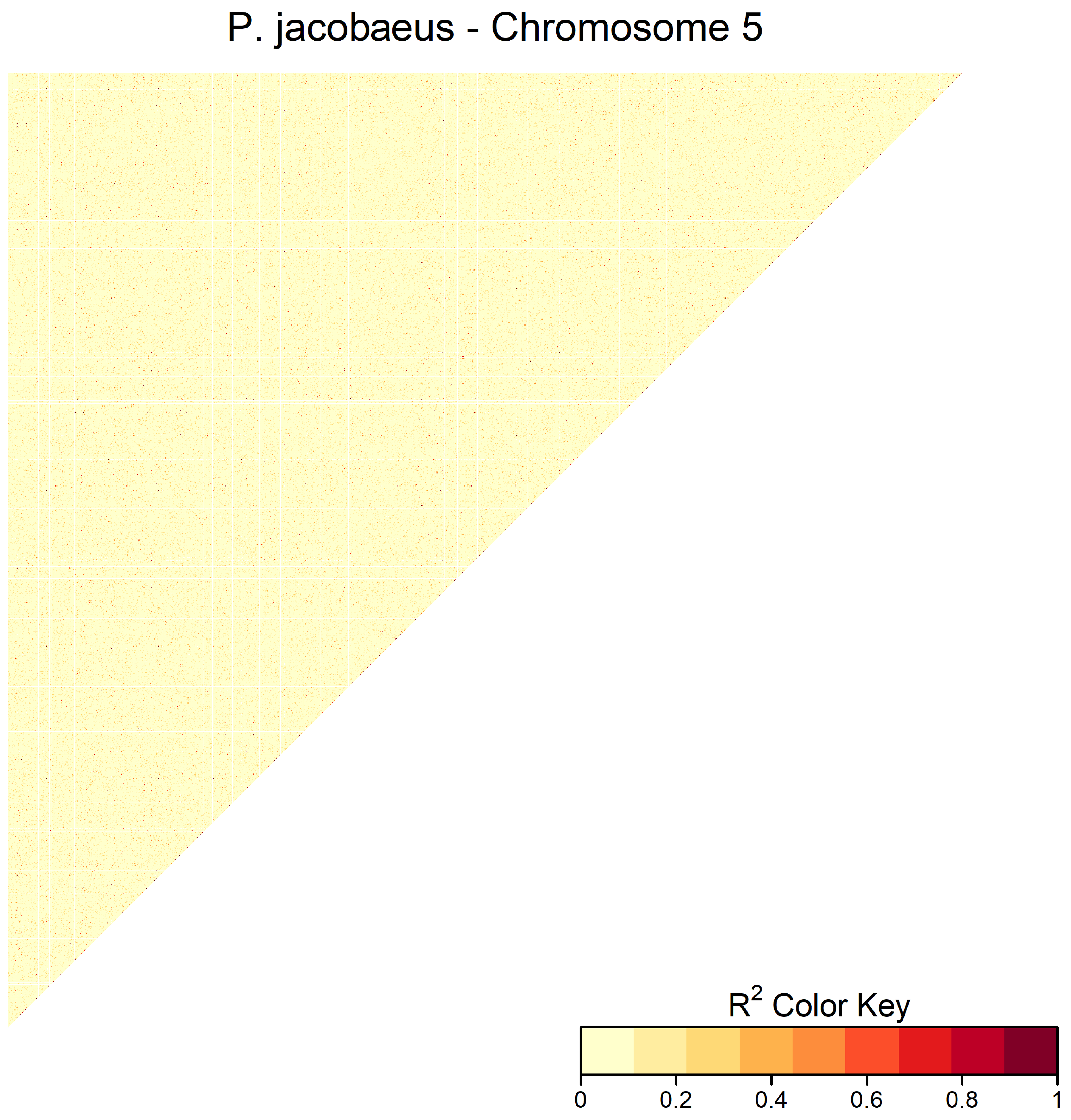
P. jacobaeus - Chromosome 5
R^2 Color Key
0
0.2
0.4
0.6
0.8
1

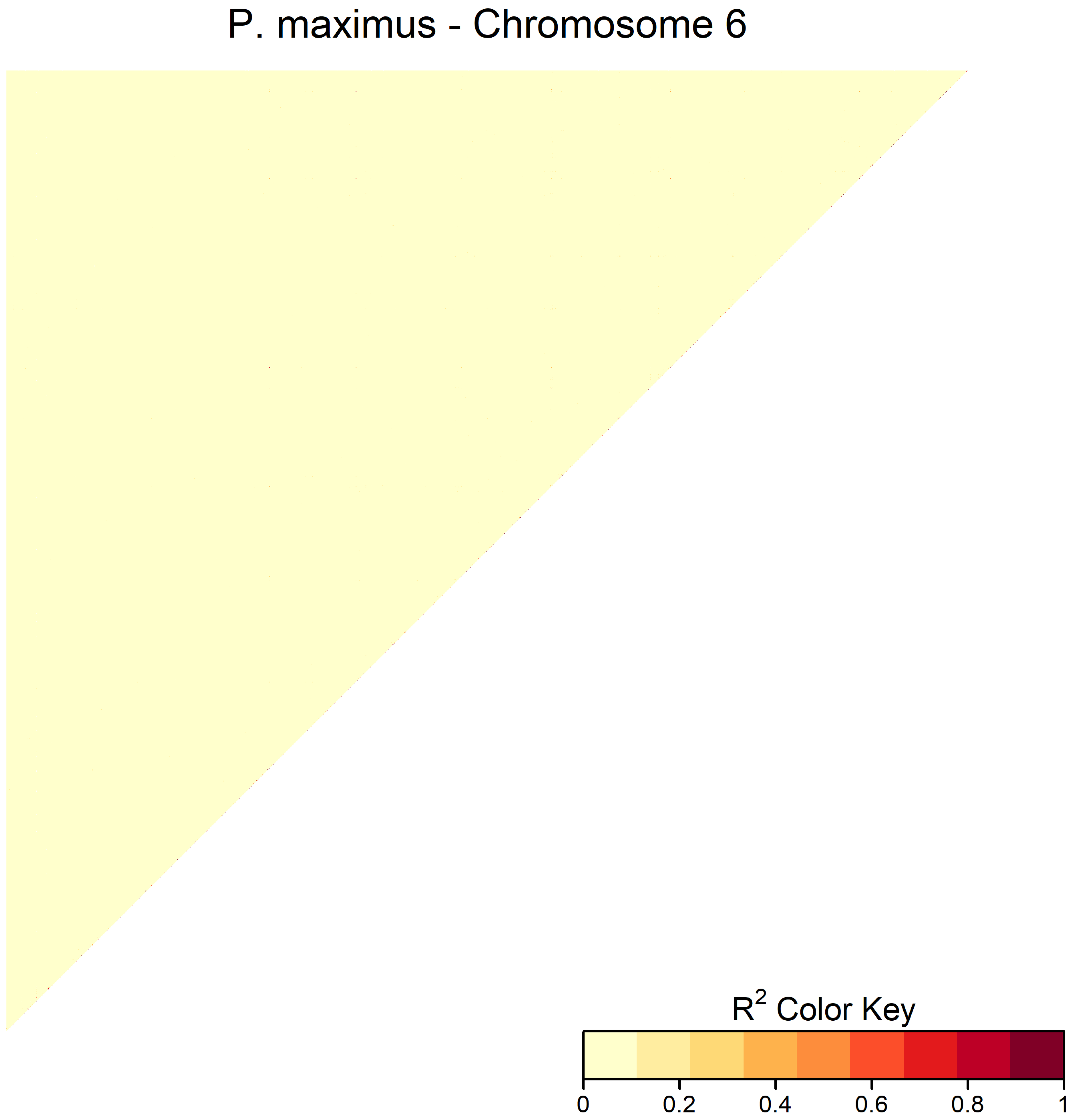
P. maximus - Chromosome 6
R2 Color Key
0
0.2
0.4
0.6
0.8
1

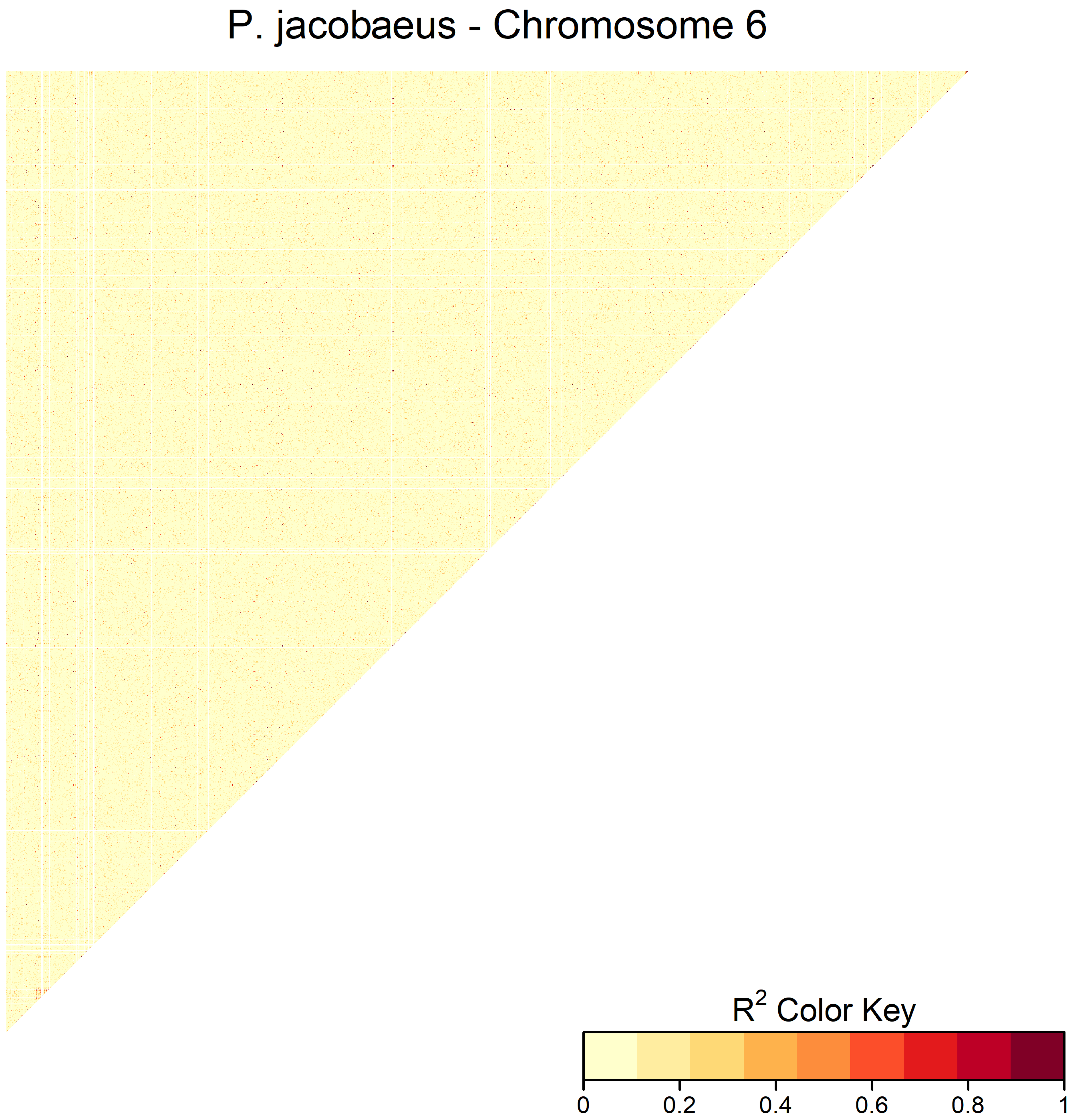
P. jacobaeus - Chromosome 6
R2 Color Key
0
0.2
0.4
0.6
0.8
1

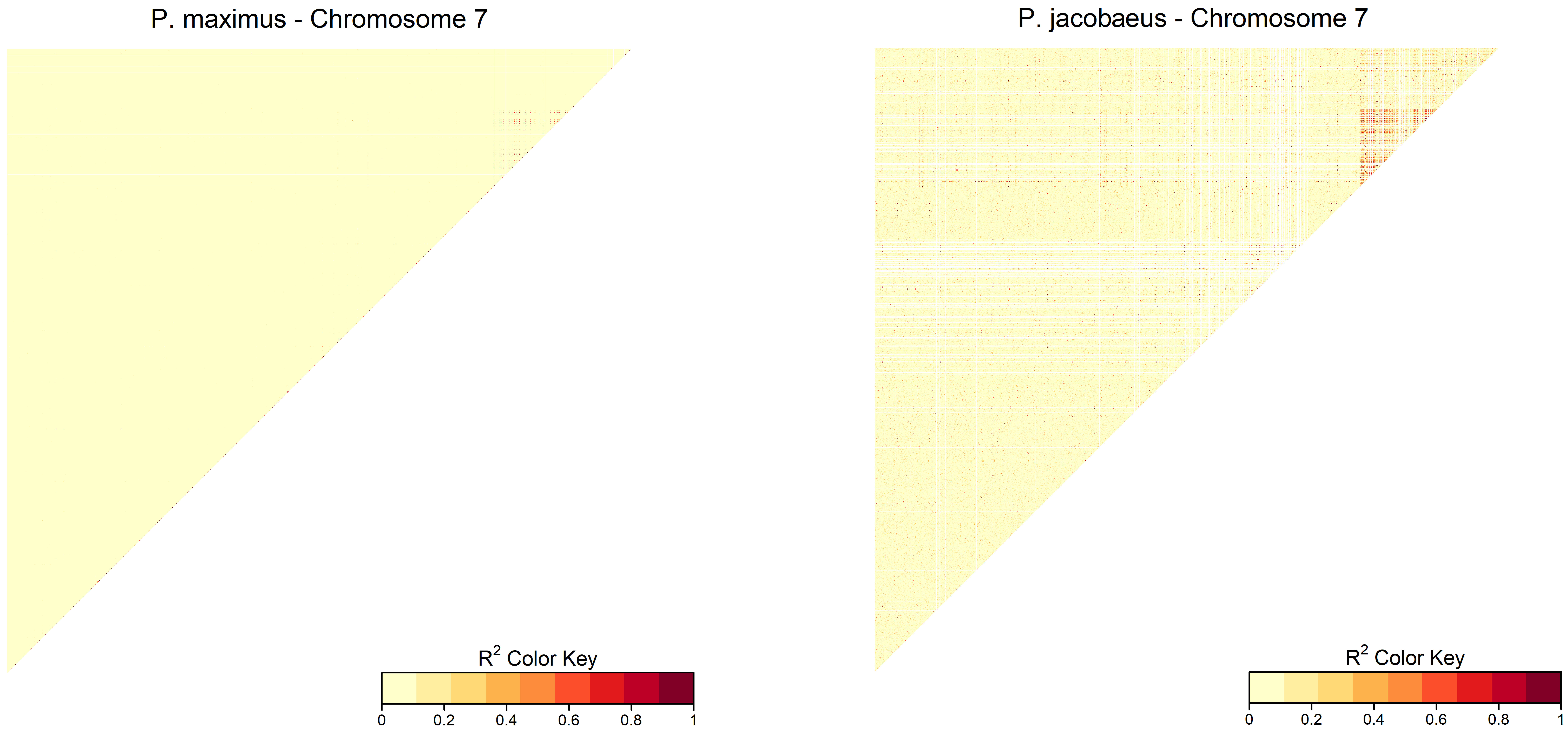
P. maximus - Chromosome 7
R^2 Color Key
0
0.2
0.4
0.6
0.8
1
P. jacobaeus - Chromosome 7
R^2 Color Key
0
0.2
0.4
0.6
0.8
1

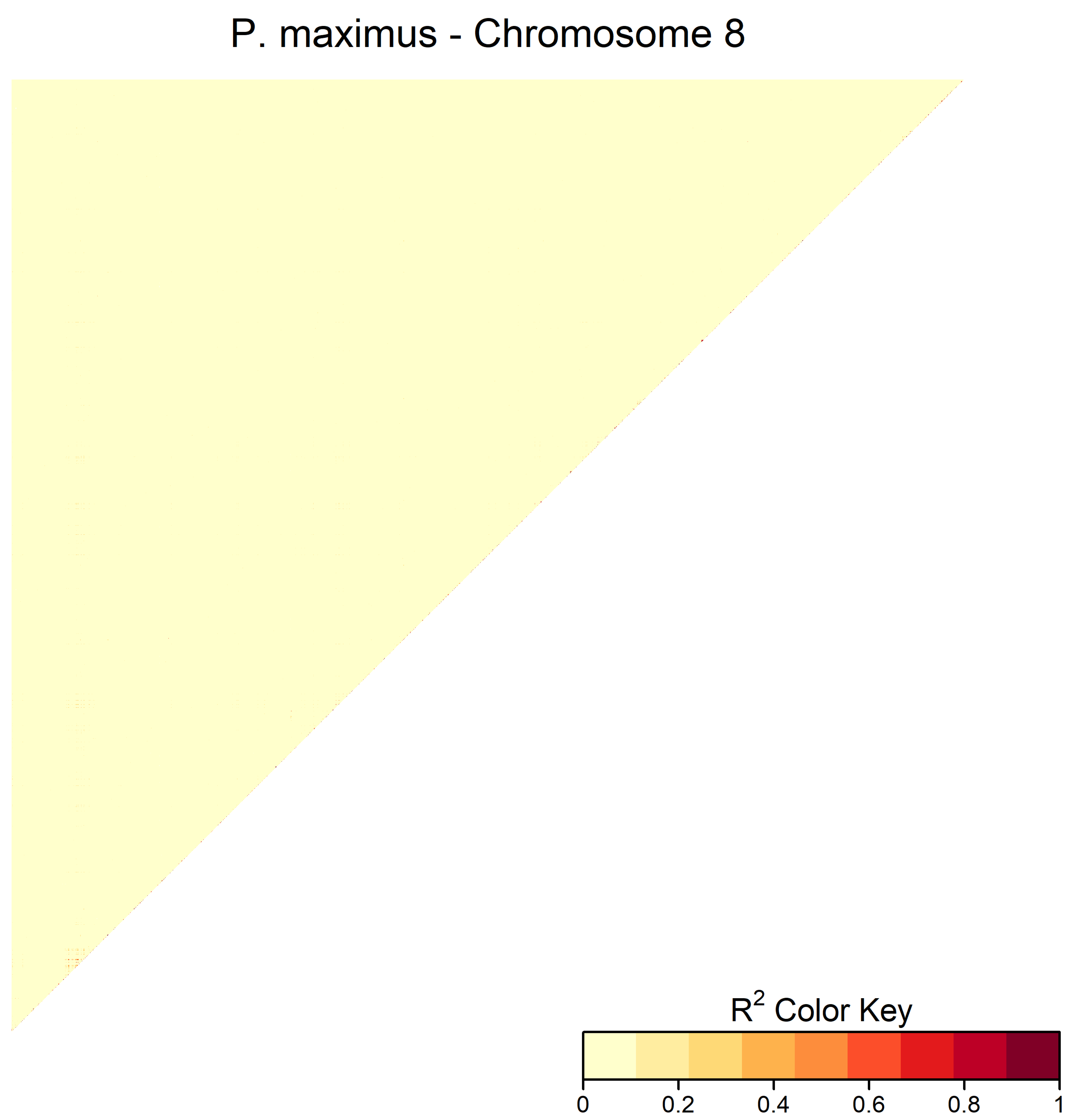
P. maximus - Chromosome 8
R^2 Color Key
0
0.2
0.4
0.6
0.8
1

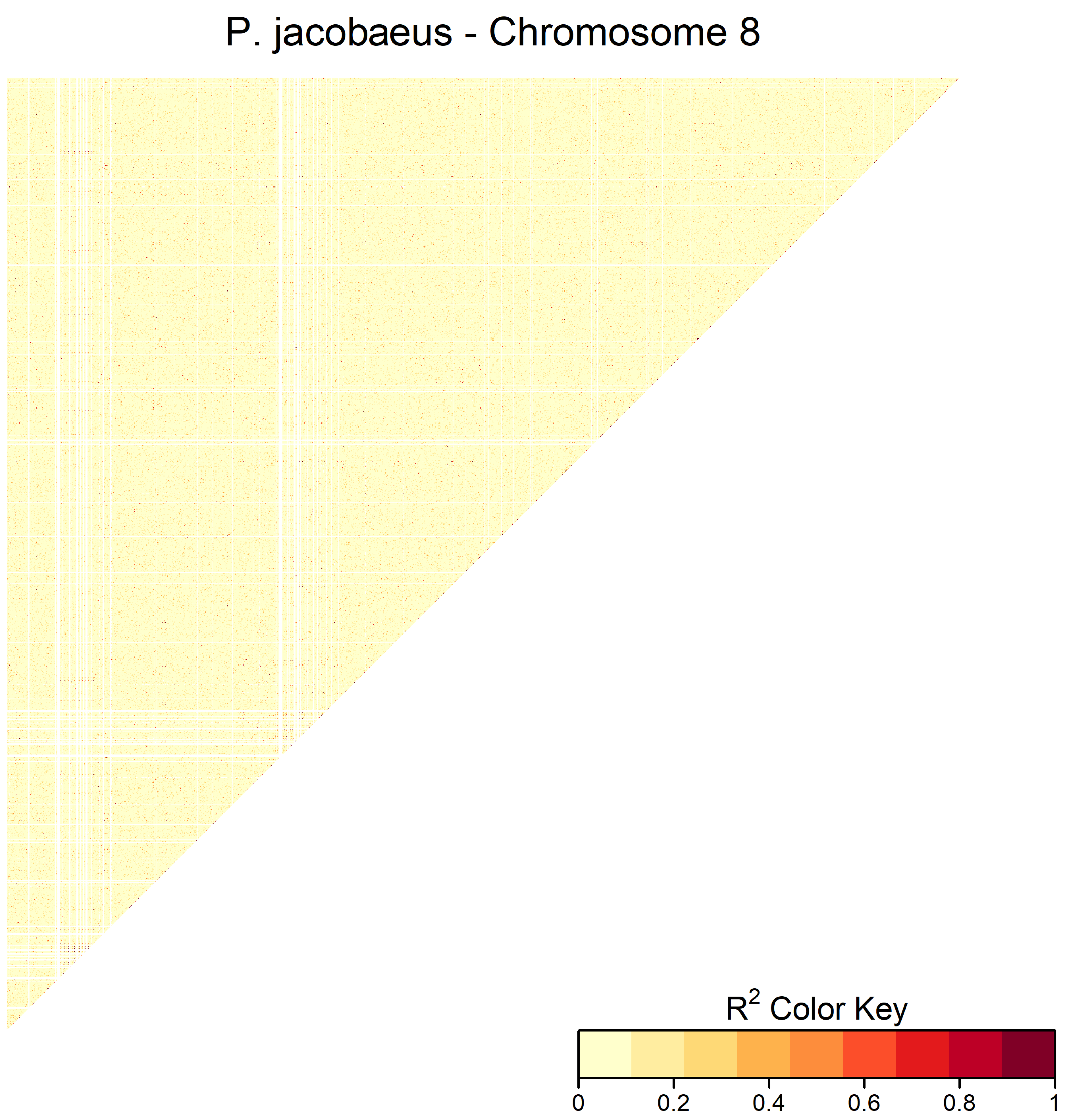
P. jacobaeus - Chromosome 8
R^2 Color Key
0
0.2
0.4
0.6
0.8
1

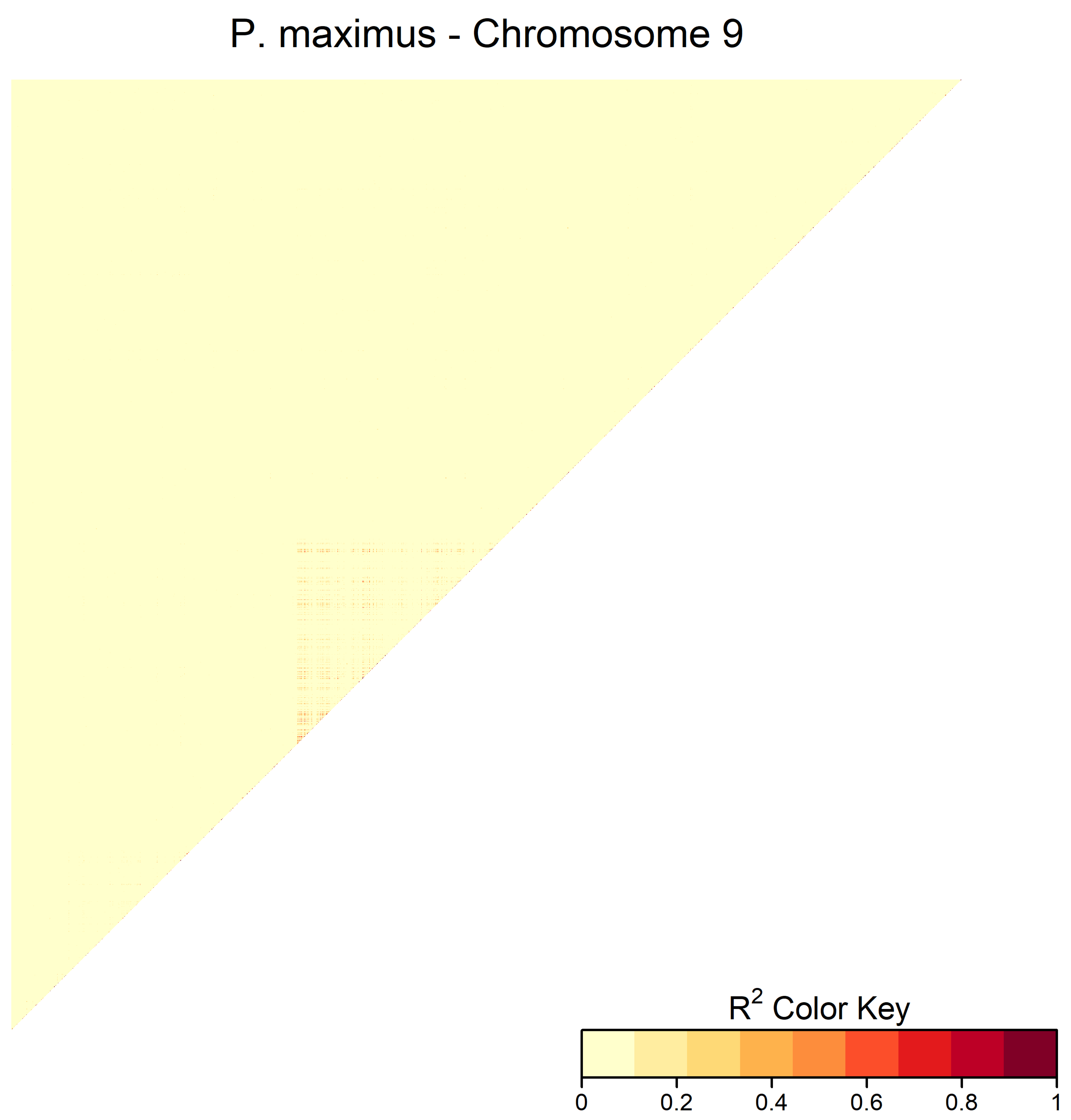
P. maximus - Chromosome 9
R^2 Color Key
0
0.2
0.4
0.6
0.8
1

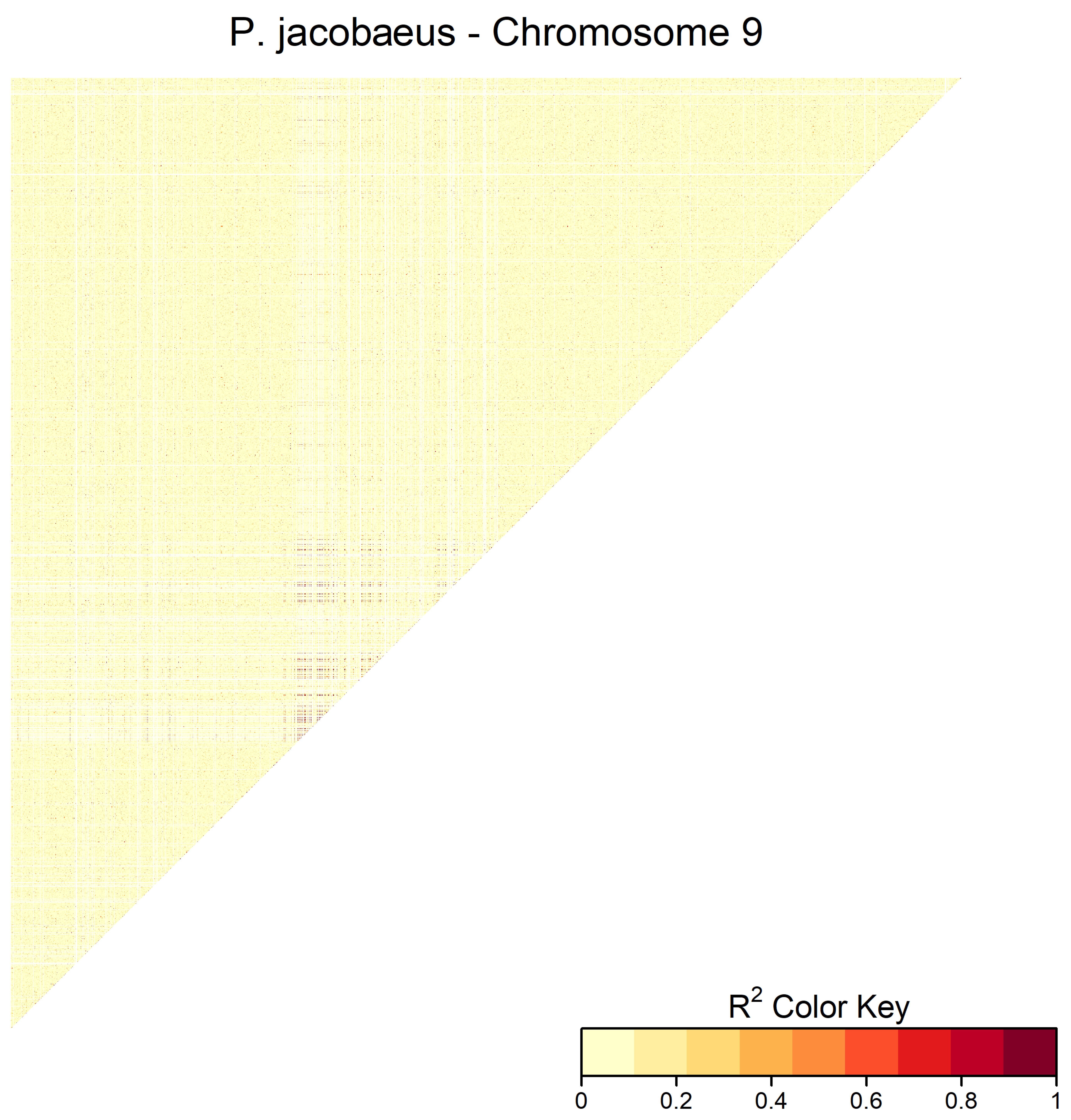
P. jacobaeus - Chromosome 9
R^2 Color Key
0
0.2
0.4
0.6
0.8
1

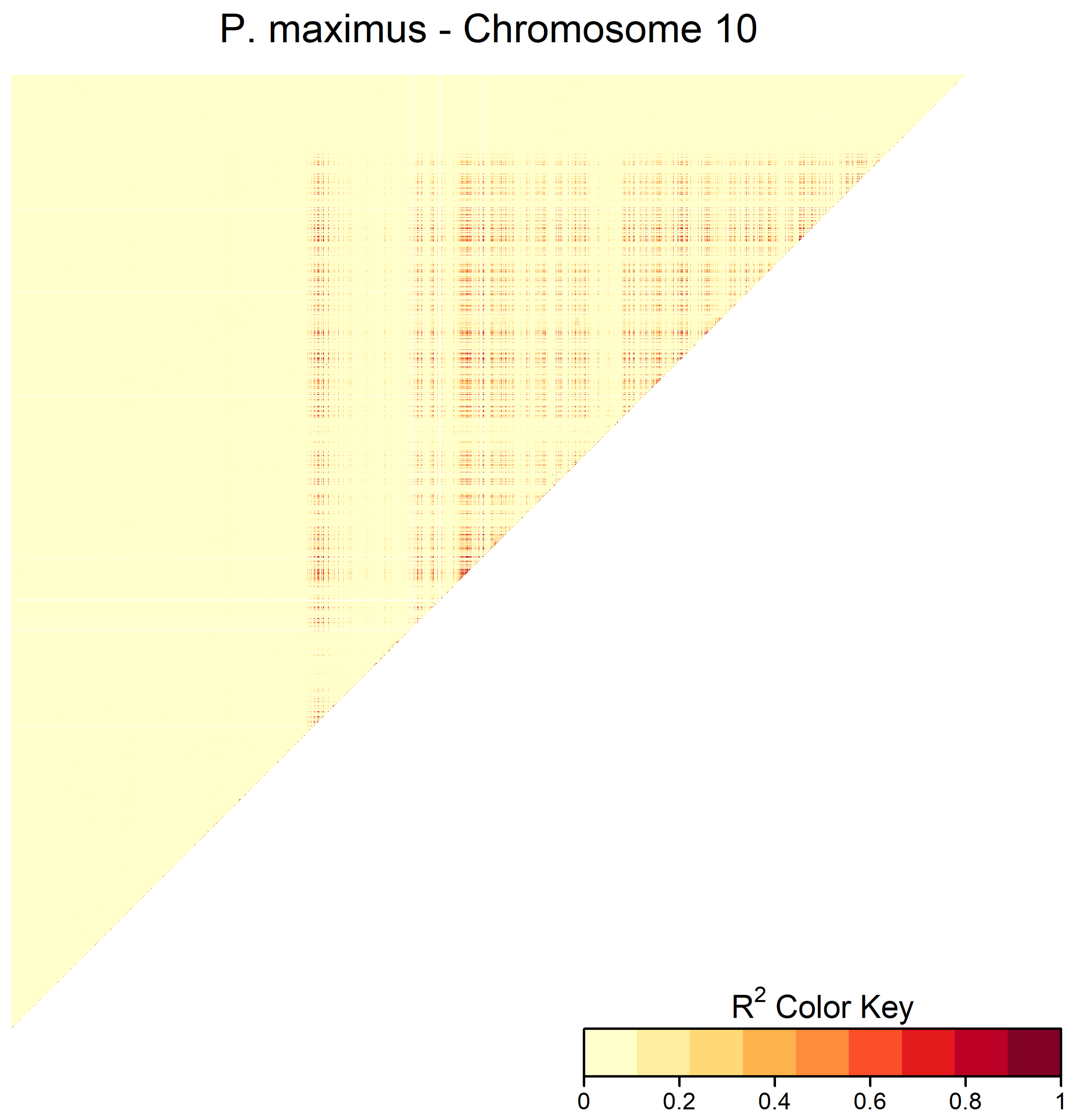
P. maximus - Chromosome 10
R² Color Key
0
0.2
0.4
0.6
0.8
1

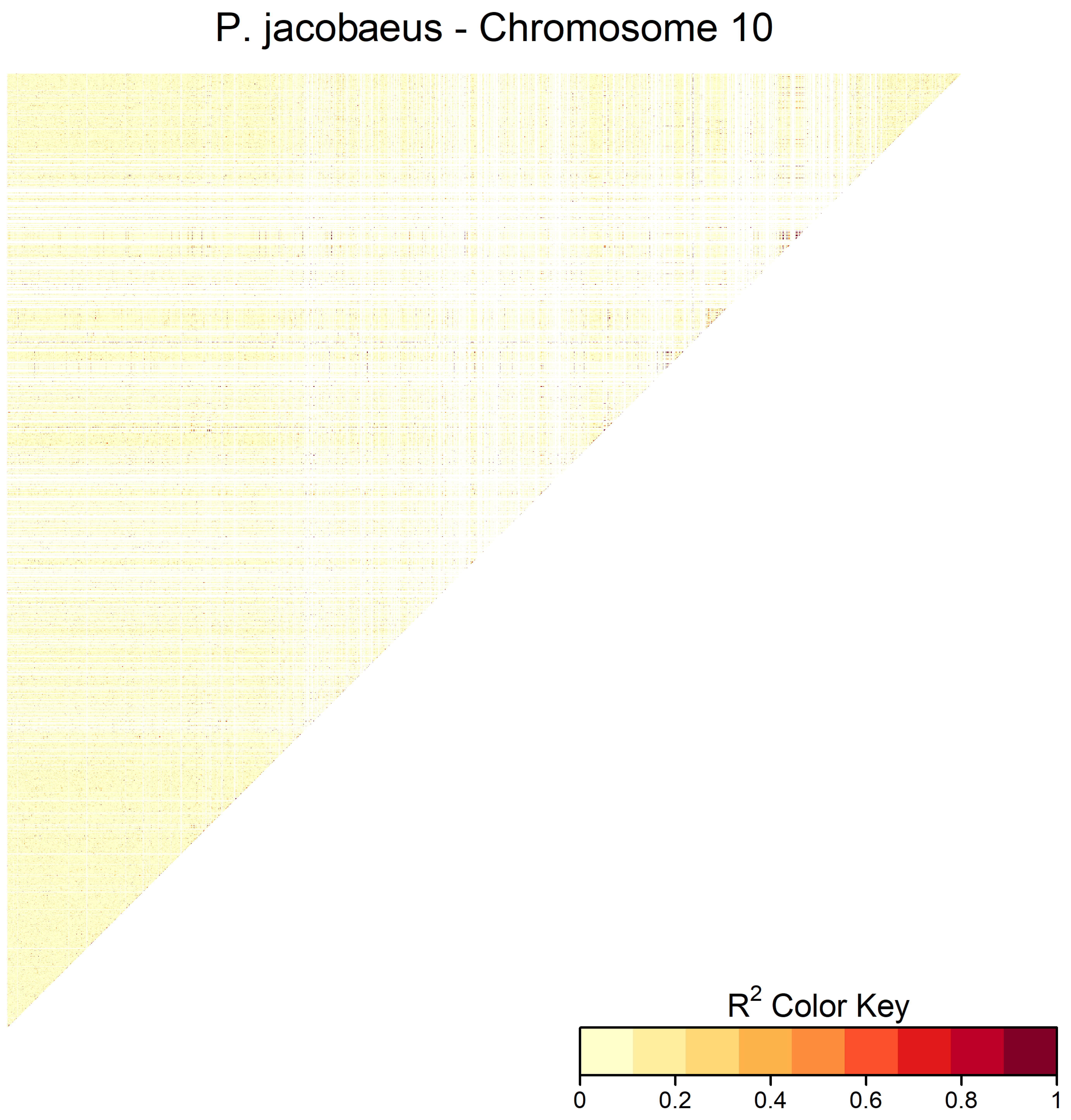
P. jacobaeus - Chromosome 10
R² Color Key
0
0.2
0.4
0.6
0.8
1

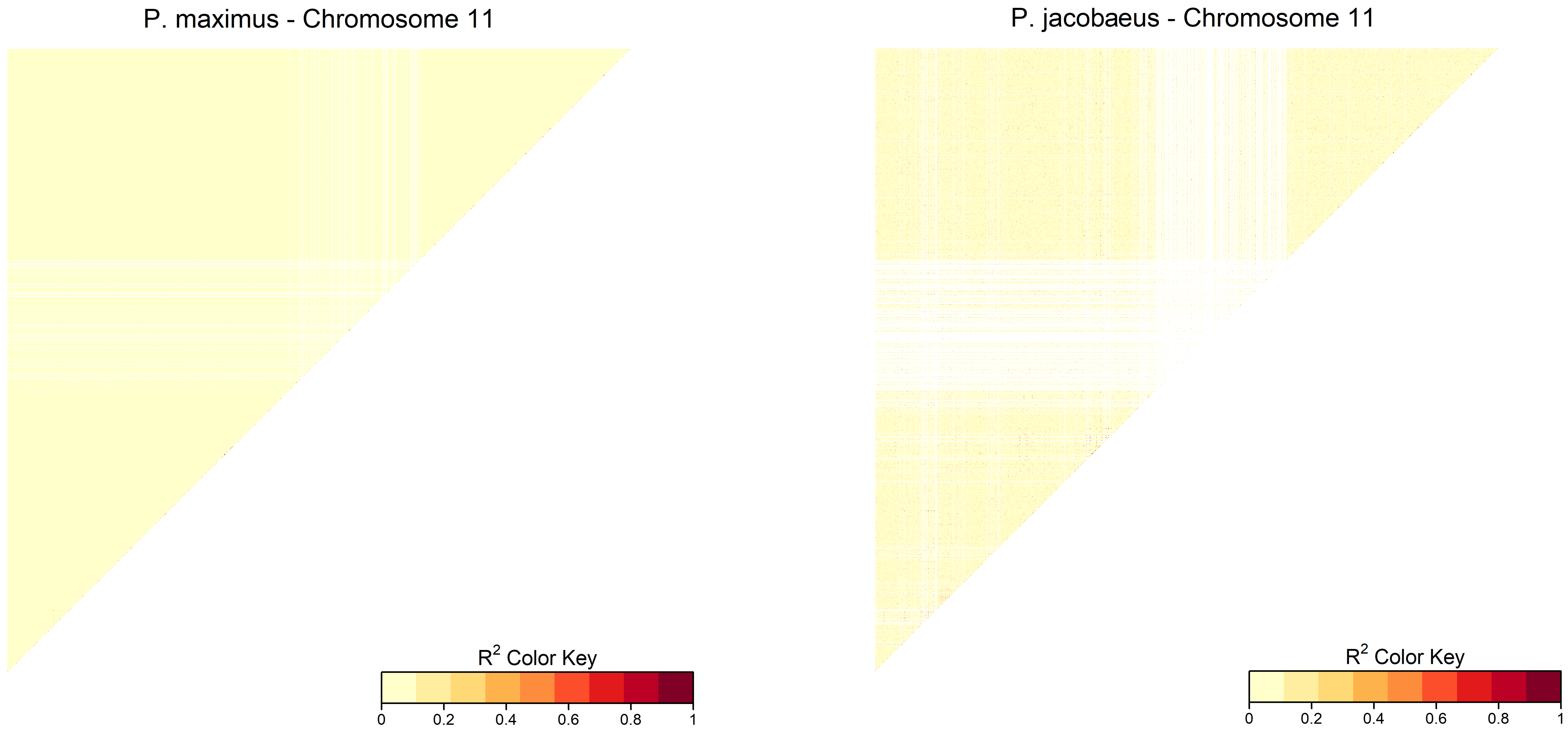

P. maximus - Chromosome 11
R^2 Color Key
0
0.2
0.4
0.6
0.8
1
P. jacobaeus - Chromosome 11
R^2 Color Key
0
0.2
0.4
0.6
0.8
1

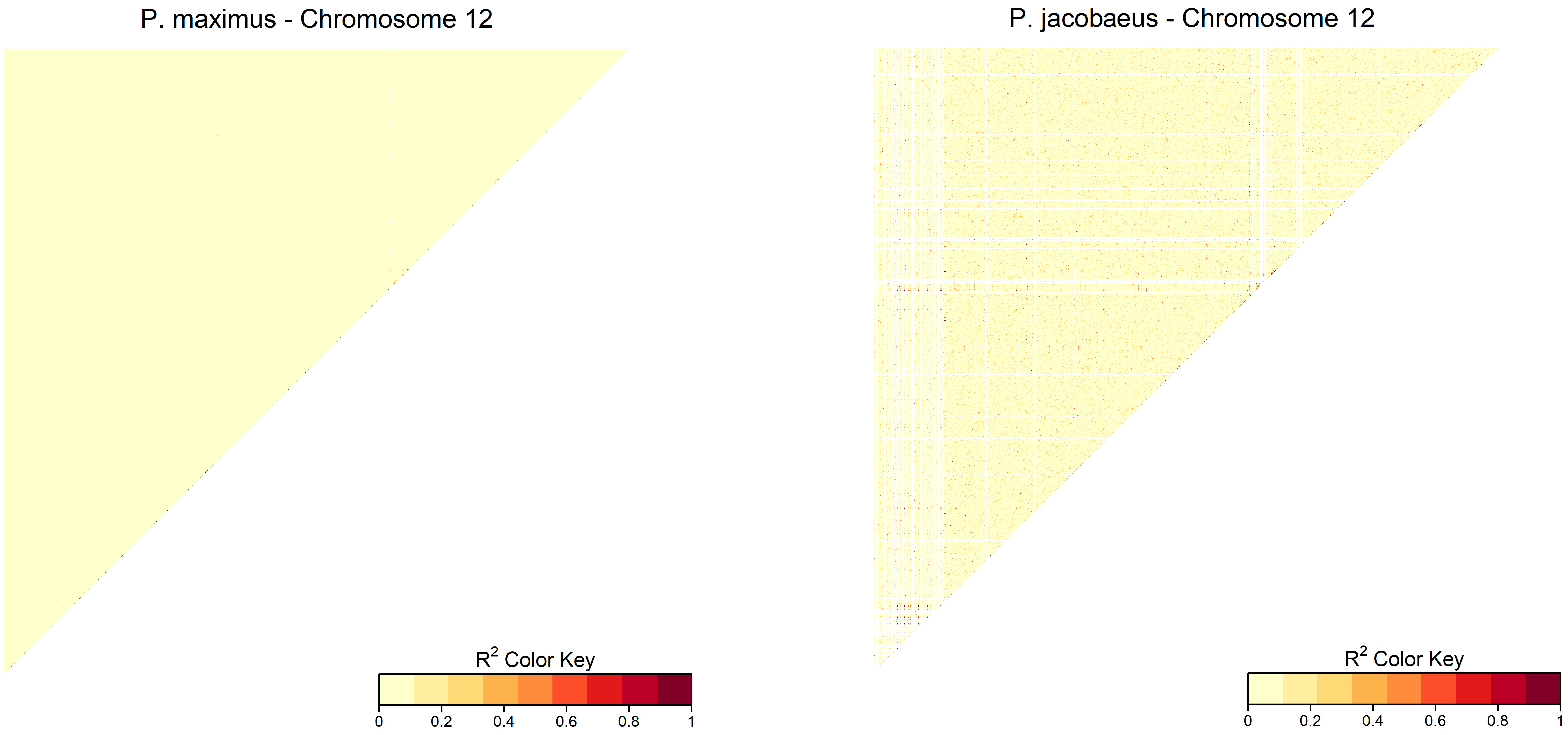
P. maximus - Chromosome 12
R2 Color Key
0
0.2
0.4
0.6
0.8
1
P. jacobaeus - Chromosome 12
R2 Color Key
0
0.2
0.4
0.6
0.8
1

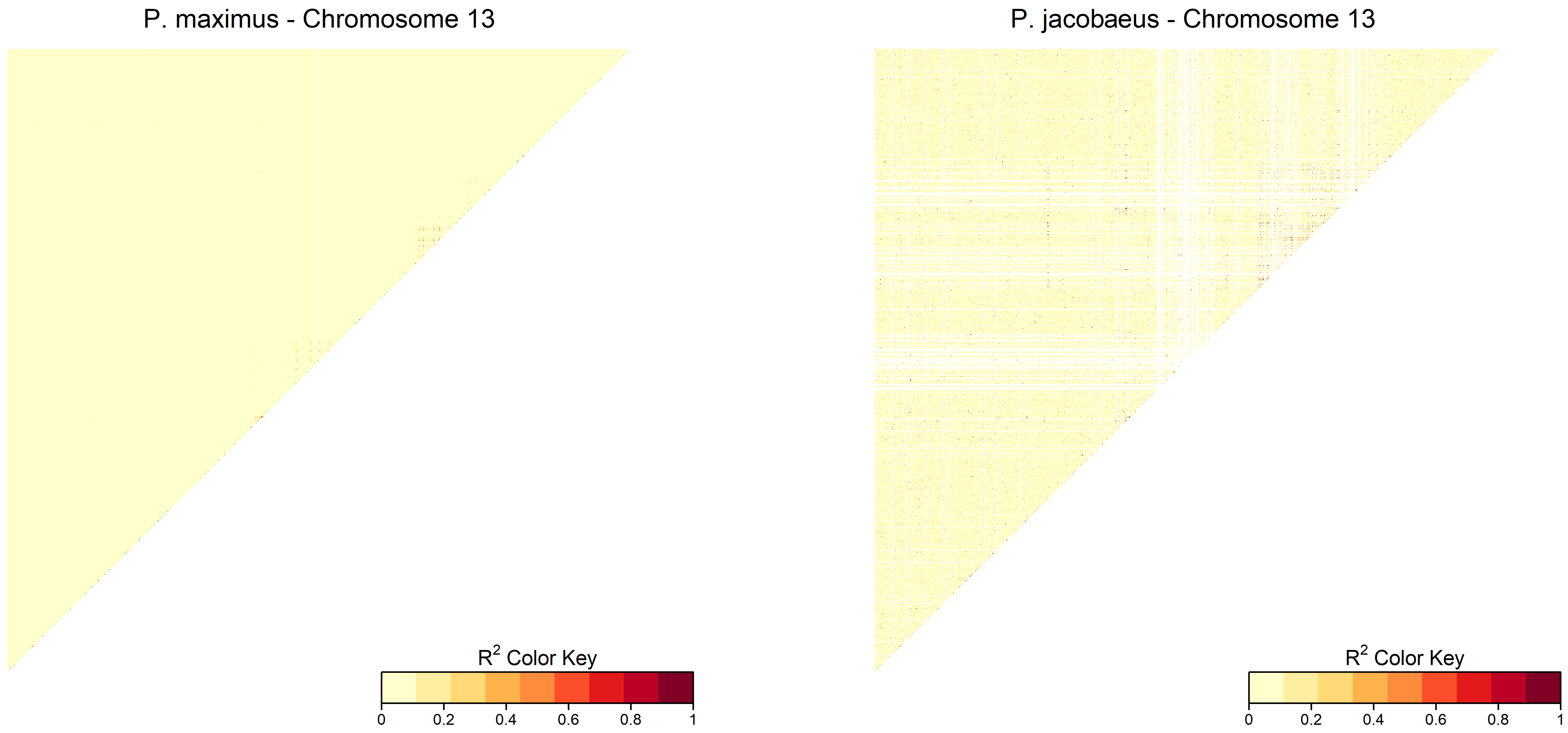
P. maximus - Chromosome 13
R^2 Color Key
0
0.2
0.4
0.6
0.8
1
P. jacobaeus - Chromosome 13
R^2 Color Key
0
0.2
0.4
0.6
0.8
1

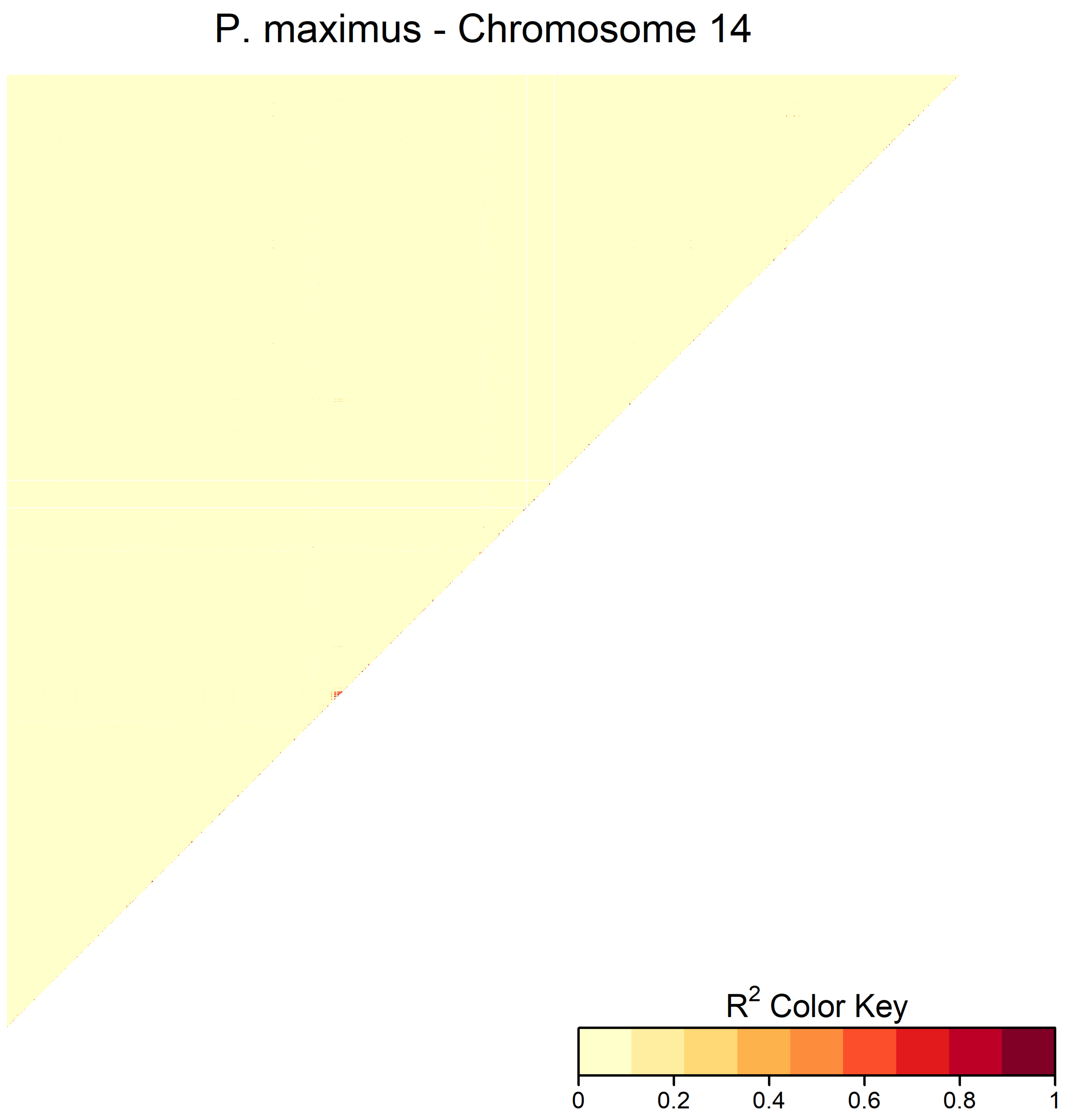
P. maximus - Chromosome 14
R^2 Color Key
0
0.2
0.4
0.6
0.8
1

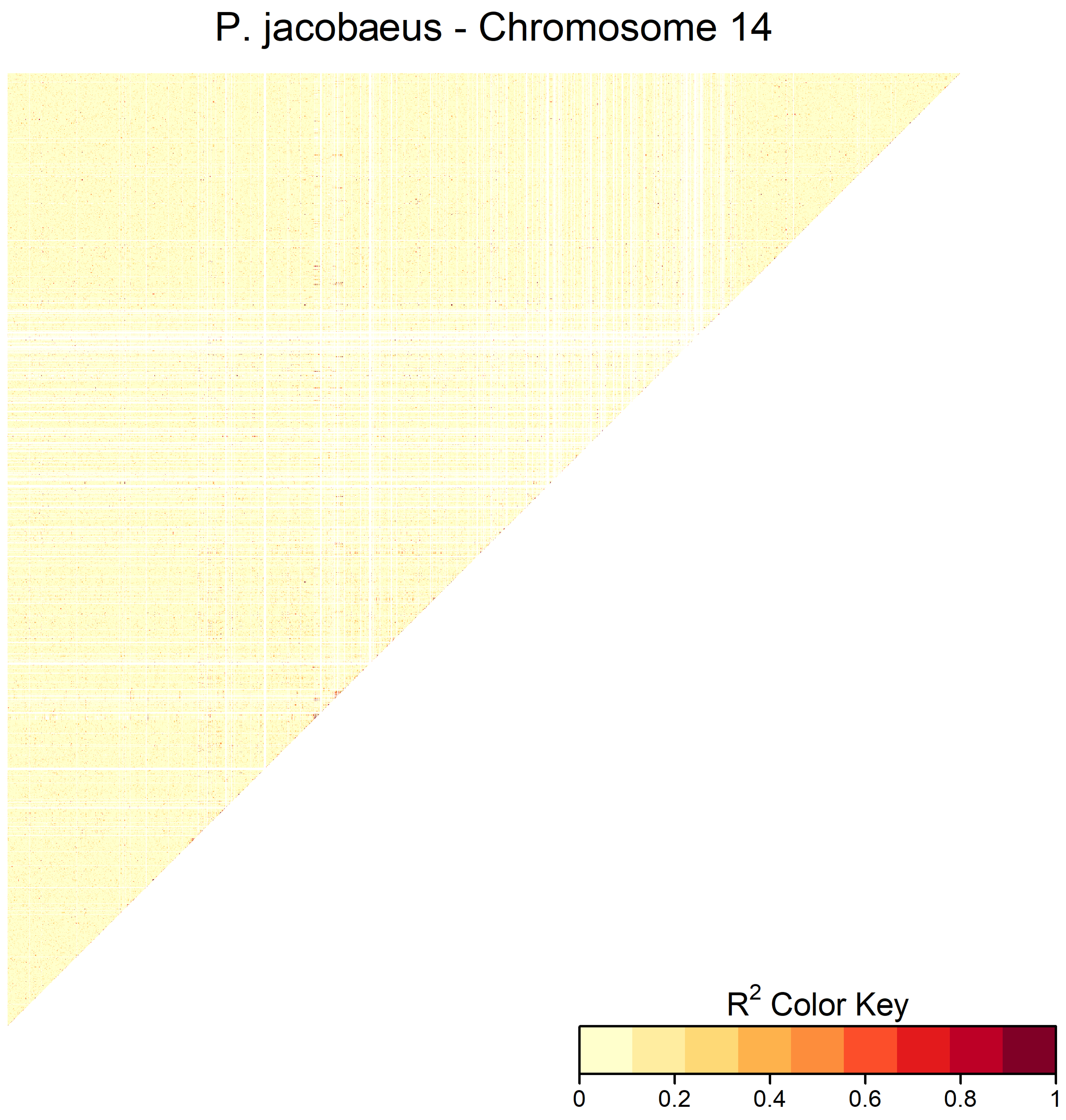
P. jacobaeus - Chromosome 14
R^2 Color Key
0
0.2
0.4
0.6
0.8
1

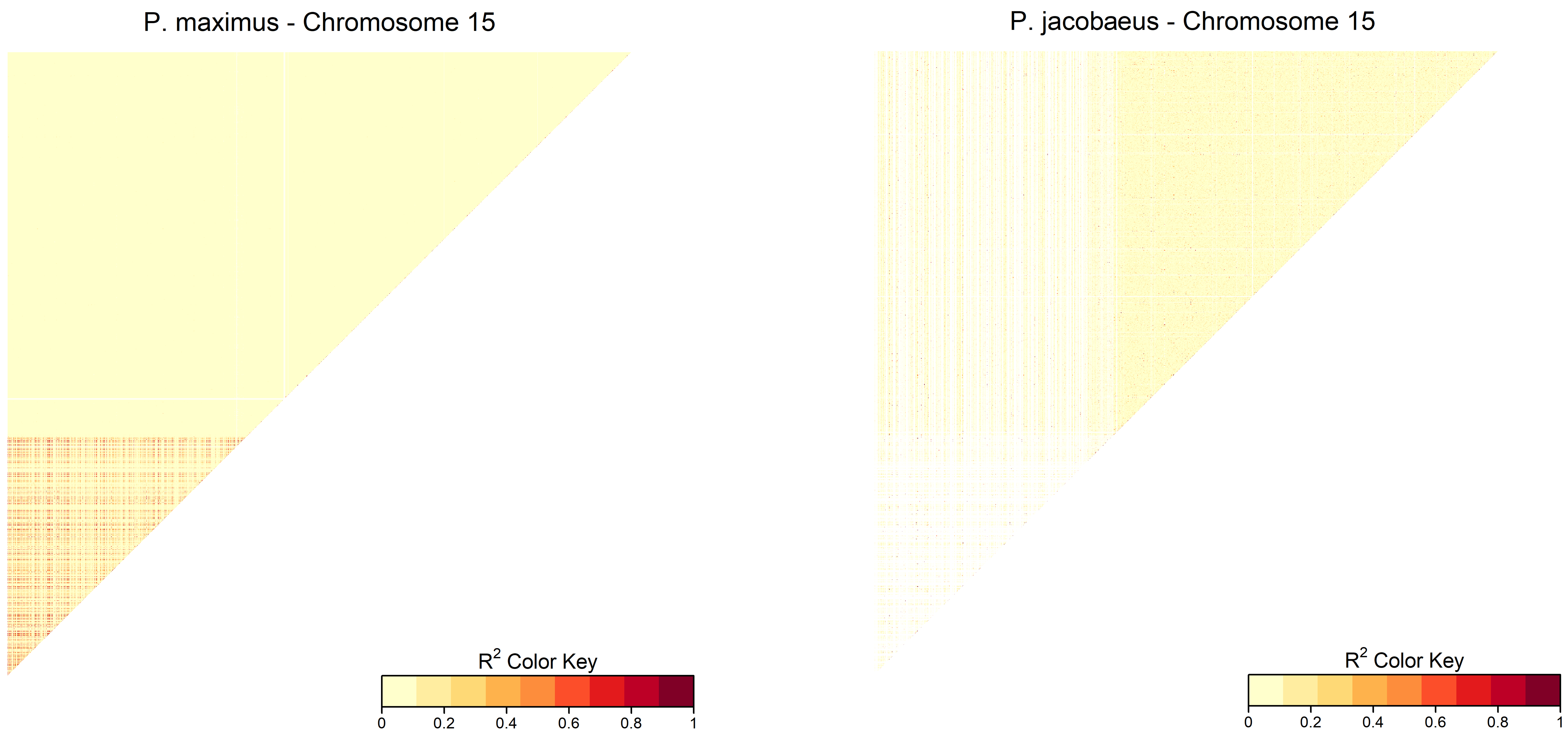

P. maximus - Chromosome 15
R2 Color Key
0 0.2 0.4 0.6 0.8 1
P. jacobaeus - Chromosome 15
R2 Color Key
0 0.2 0.4 0.6 0.8 1

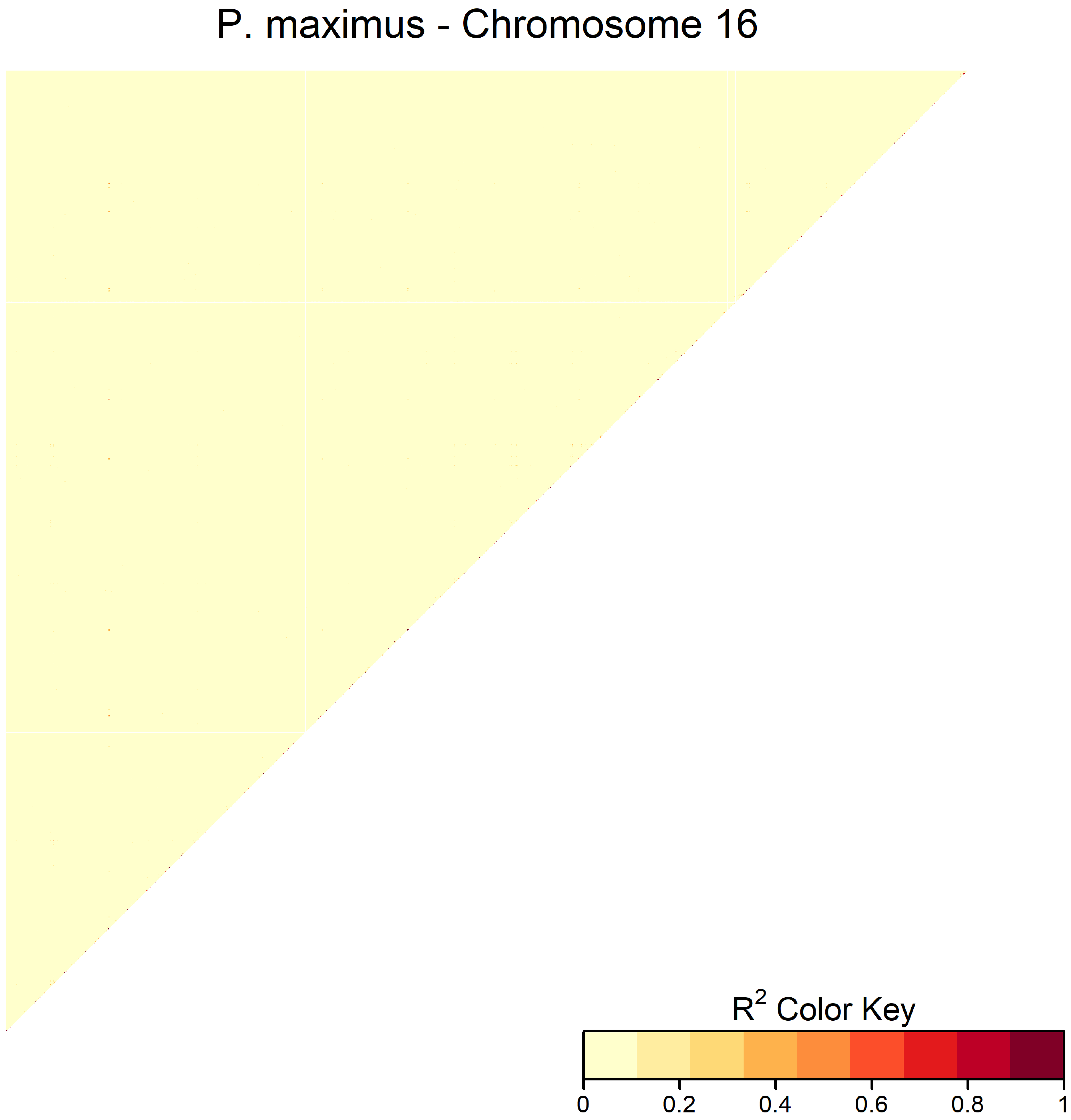
P. maximus - Chromosome 16
R2 Color Key
0
0.2
0.4
0.6
0.8
1

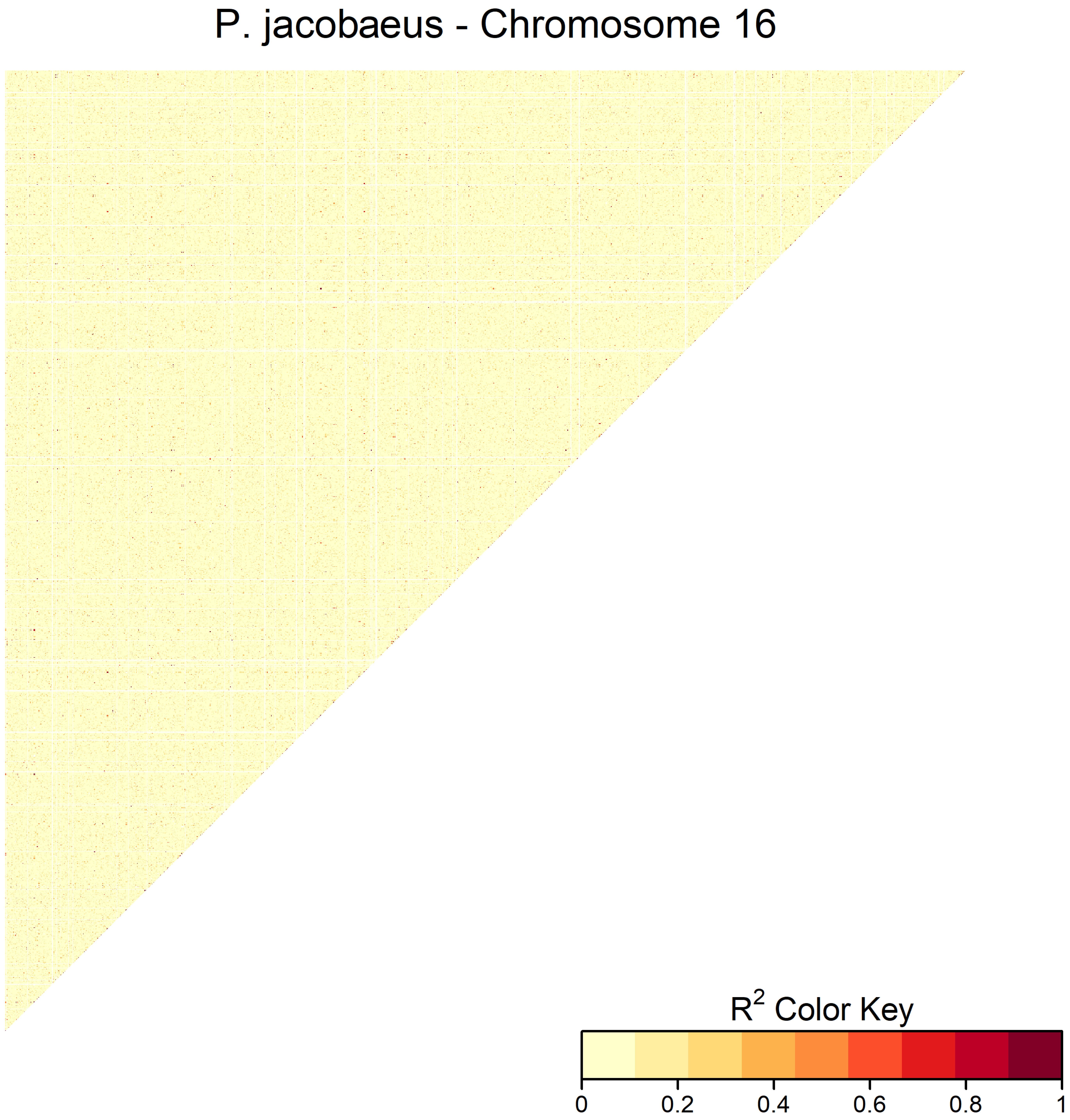
P. jacobaeus - Chromosome 16
R2 Color Key
0
0.2
0.4
0.6
0.8
1

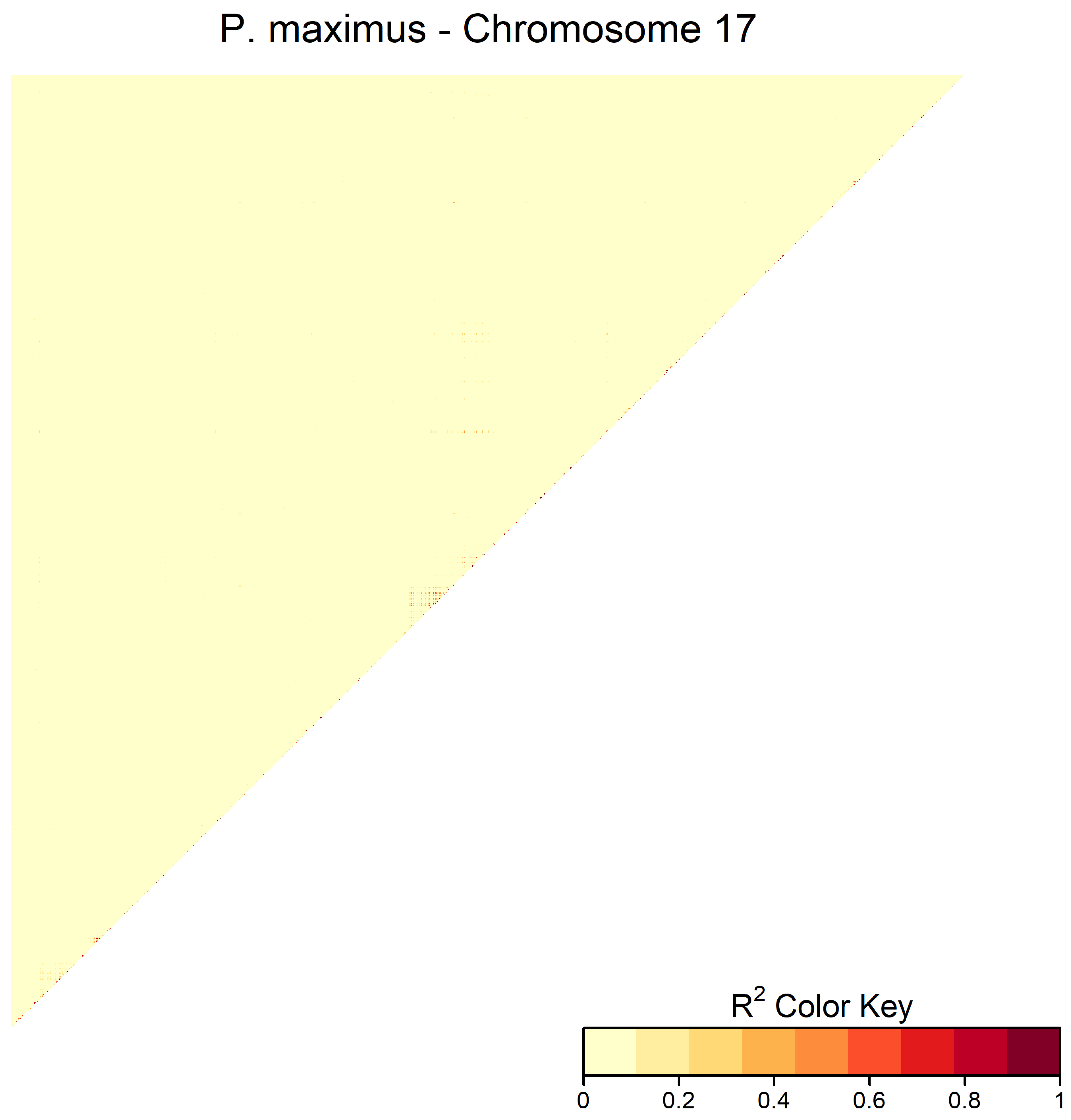
P. maximus - Chromosome 17
R^2 Color Key
0
0.2
0.4
0.6
0.8
1

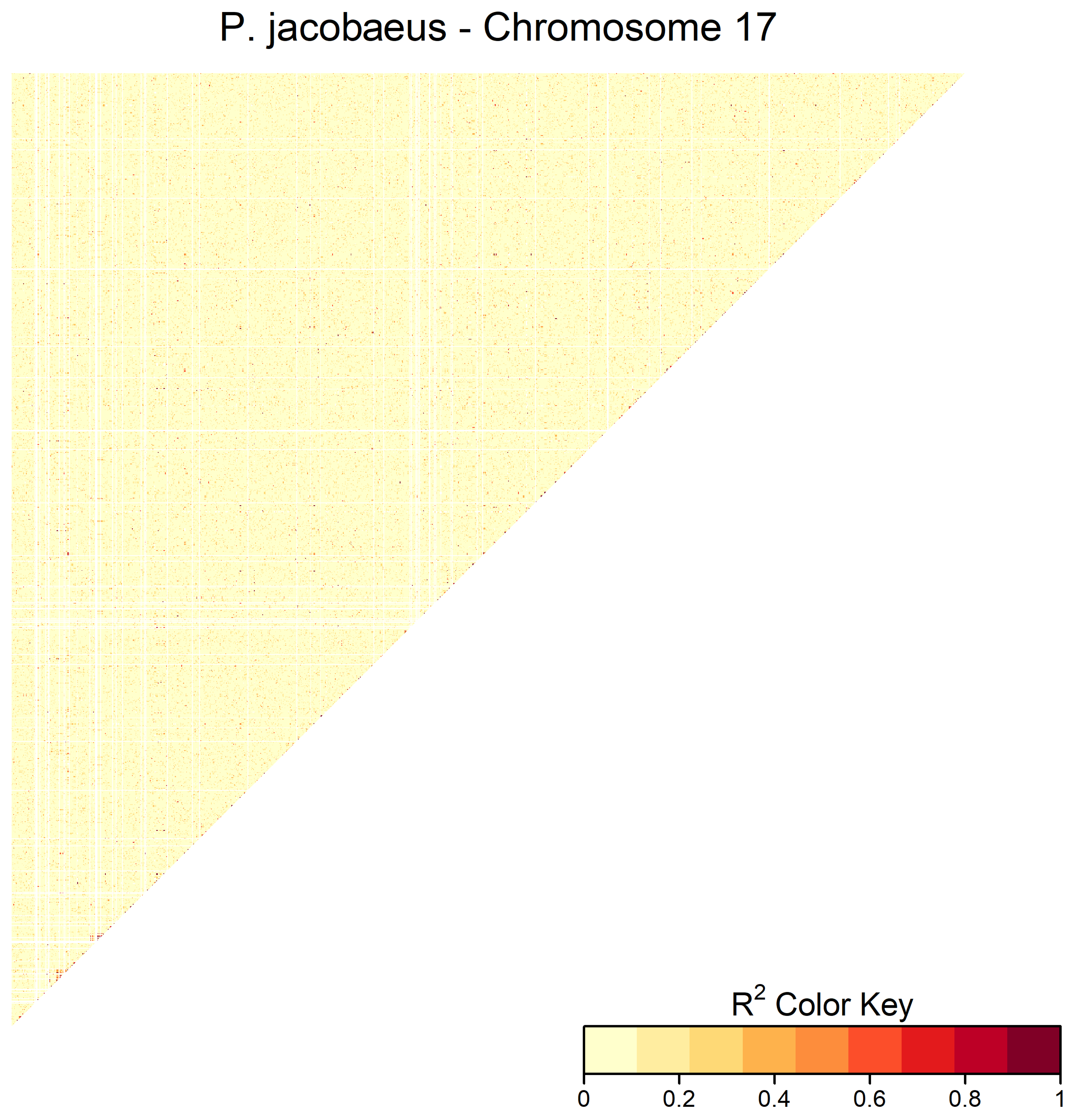
P. jacobaeus - Chromosome 17
R^2 Color Key
0
0.2
0.4
0.6
0.8
1

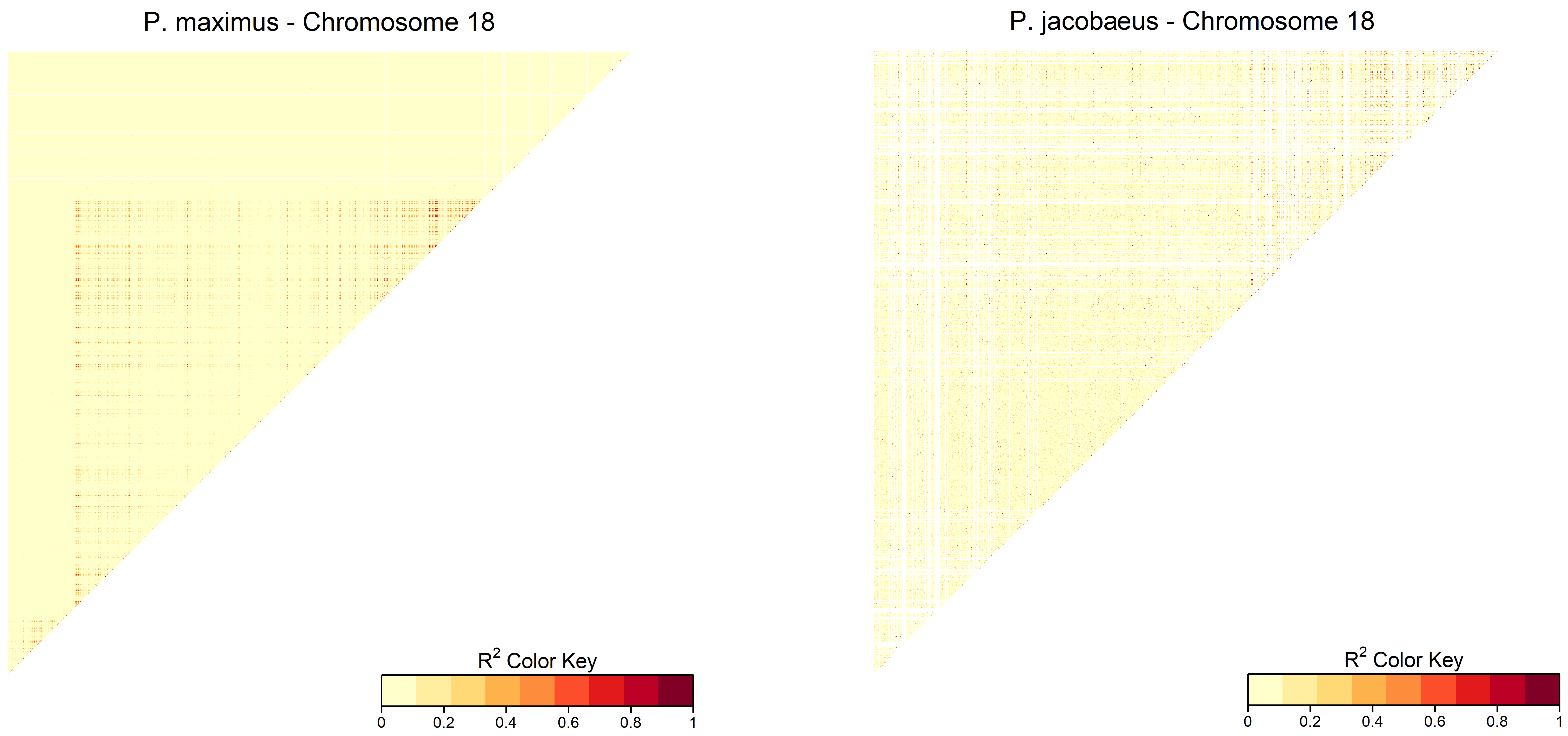

P. maximus - Chromosome 18
R2 Color Key
0
0.2
0.4
0.6
0.8
1
P. jacobaeus - Chromosome 18
R2 Color Key
0
0.2
0.4
0.6
0.8
1

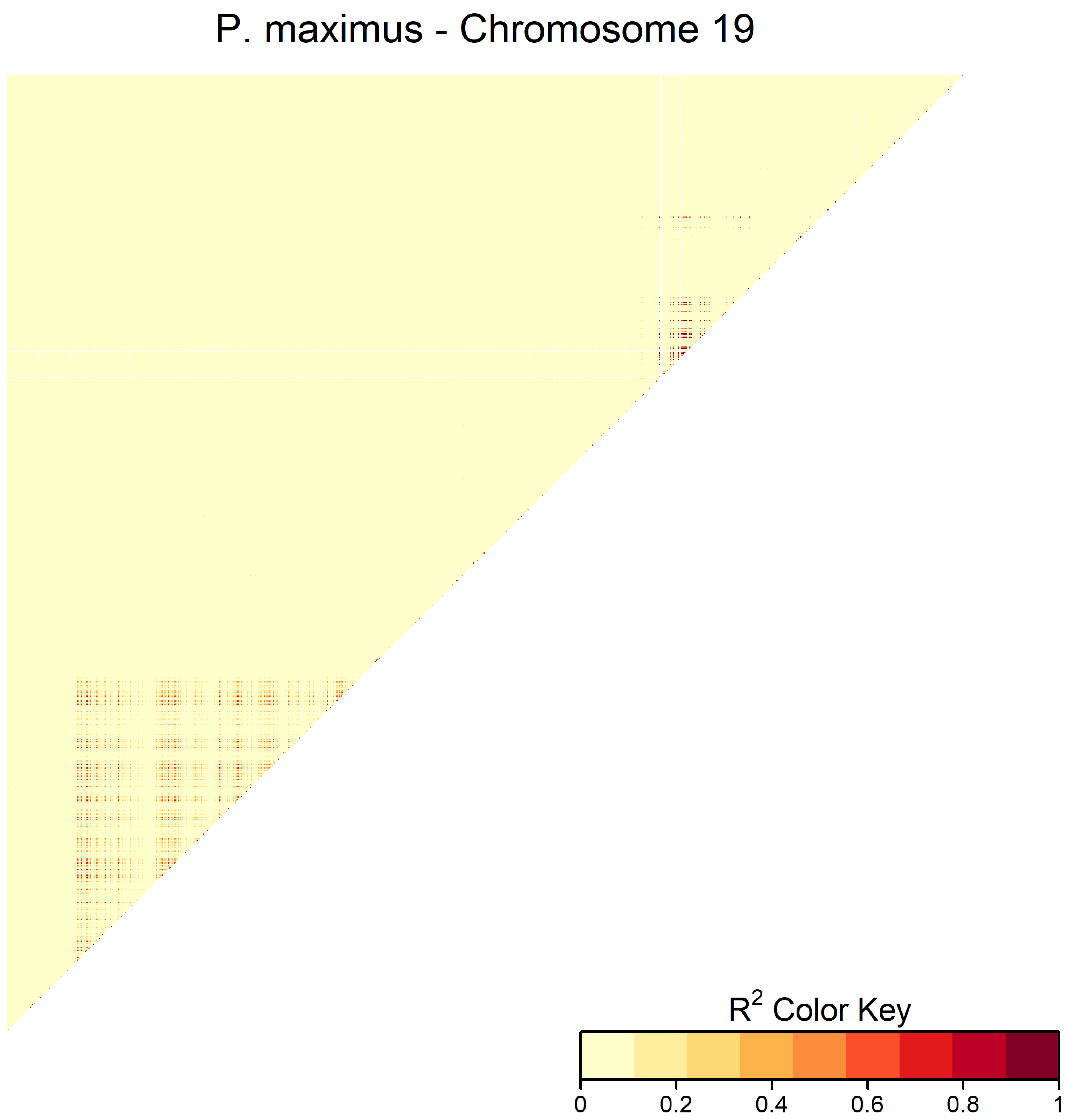
P. maximus - Chromosome 19
R^2 Color Key
0
0.2
0.4
0.6
0.8
1

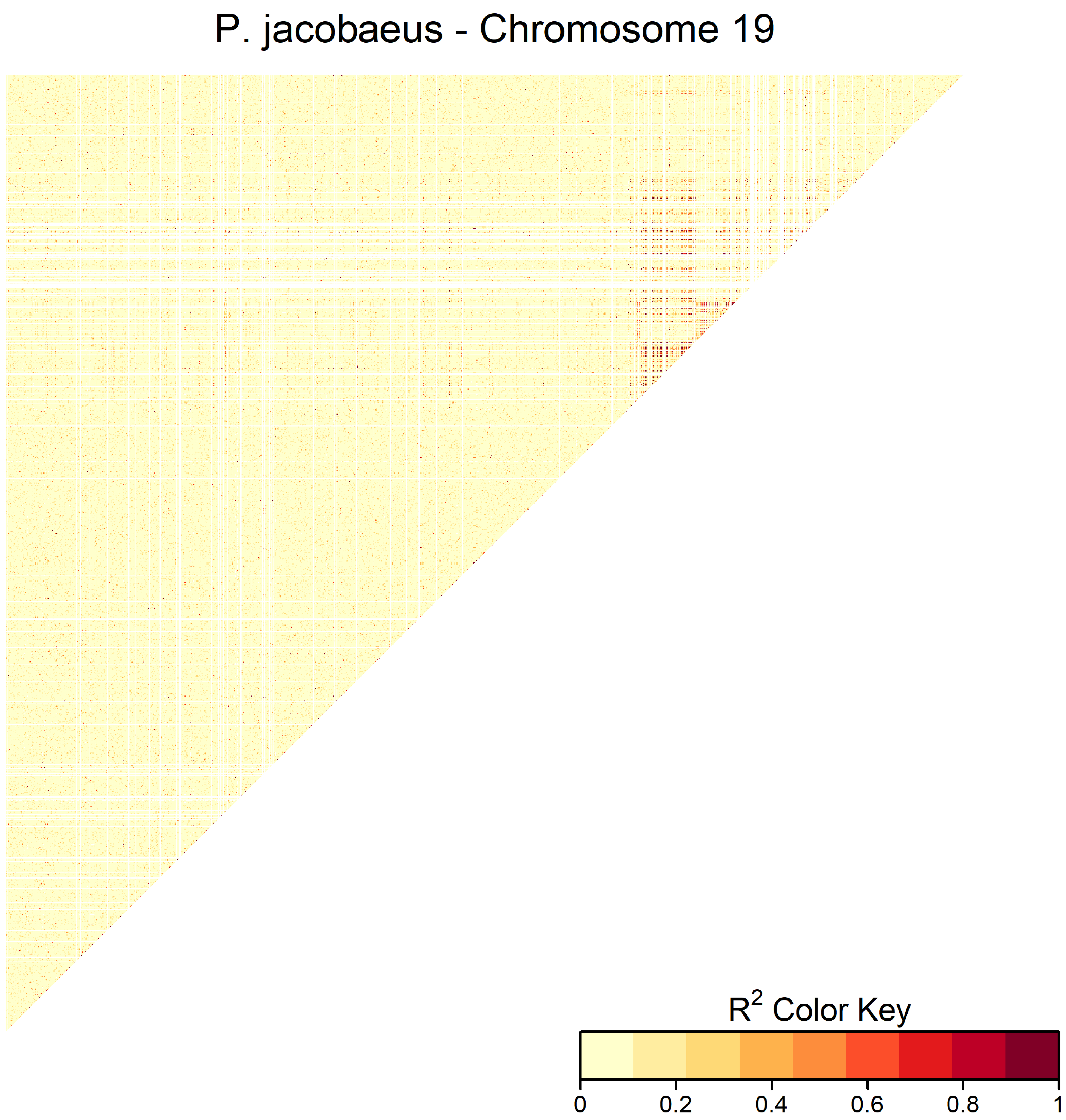
P. jacobaeus - Chromosome 19
R^2 Color Key
0
0.2
0.4
0.6
0.8
1
