# Supplementary material for: Heterogeneous Genomic Divergence Landscape in Two Commercially Important European Scallop Species
Source: Genes (Basel). 2022 Dec 21;14(1):14. doi: 10.3390/genes14010014 (PMC9858869; doi:10.3390/genes14010014)
Supplement: Supplementary file 1 [file Supplementary material captions.pdf › Supplementary material captions.pdf]

Supplementary Figures and Tables legends for the article "Heterogeneous genomic divergence landscape in two commer-cially important European scallop species" by David L. J. Vendrami, Joseph I. Hoffman and Craig S. Wilding.

Figure S1: Schematic showing tested alternative demographic models. In all five models, the *P. maximus* and *P. jacobaeus* lineages diverged T_S generations ago. Subsequently, (a) depicts the SI model where no gene flow occurs after the split between the two lineages; (b) depicts the IM model where continuous gene flow occurs after the split between the two lineages; (c) depicts the AM model where initial gene flow occurs after the split between the two lineages, until T_1 generations ago; (d) depicts the SC model, where gene flow occurs in the form of secondary contact starting T_1 generations ago until present; and (e) depicts the SCS model where gene flow occurs in the form of secondary contact starting T_1 generations ago until T_2 generations ago. In panels (b - d) *mjm* and *mmj* represent the migration rates from the *P. jacobaeus* lineage to the *P. maximus* lineage and vice versa respectively. All models assumes constant population size for all lineages and effective population sizes are indicated as N_{pma}, for the *P. maximus* lineage, as N_{pja}, for the *P. jacobaeus* lineage, and as N_{anc}, for the ancestral *Pecten* lineage predating the split between the two species. Shaded red areas in the schematic represent the time windows during which gene flow occurred.

Figure S2: F_{ST} values calculated between *P. maximus* and *P. jacobaeus* samples within 100kb sliding windows using SNP datasets that were generated using different filtering strategies. Panel (a) shows the results already presented in the main manuscript (see main manuscript for details on filtering criteria). Panel (b) shows results based on a dataset where SNPs genotyping rate was assessed separately for *P. maximus* and *P. jacobaeus*. SNPs were removed when their genotyping rate was below 80% in both species. This resulted in the addition of 33 SNPs that were genotyped uniquely in *P. jacobaeus*. Panel (c) shows the results based on a SNP dataset that was not filtered based on MAF, while panel (d) shows the results based on a SNP dataset where a MAF cutoff of 1% was applied. Panel (e) and (f) show the results based on SNP datasets where SNPs that showed deviations from Hardy-Weinberg equilibrium in all and half, respectively, of the sampled populations were excluded. In all panels, each point represents the F_{ST} value of a given window located on a given chromosome, as indicated on the x axis. Light and dark grey blocks correspond to the 19 chromosomes. The red dashed lines represent thresholds corresponding to the 95th percentile FST values.

Figure S3: Nucleotide diversity (π) calculated within 100kb windows. Panel (a) and (b) show results for *P. maximus* and *P. jacobaeus* respectively. Each point represents the nucleotide diversity value within a given window located within a given chromosome, as indicated on the x axis. Light and dark grey blocks correspond to the 19 different chromosomes. Red points refer to windows whose F_{ST} value was above the 95th percentile.

Figure S4: Box plots showing levels of genetic divergence between *P. maximus* and *P. jacobaeus* samples within highly divergent genomic windows ("islands") and the remaining genomic windows ("non-islands"). Panel (a) shows results based on the relative measure of genetic divergence F_{ST}, while panel (b) shows results based on the absolute measure of genetic divergence (d_{xy}).

Figure S5: Plots of linkage disequilibrium (r^2) against physical distance between SNPs in *P. maximus* (panels a and c) and *P. jacobaeus* (panels b and d). In panels (a) and (b) LD was calculated using all SNPs genotyped in *P. maximus* and *P. jacobaeus*. In panels (c) and (d) LD was calculated using only SNPs located within highly divergent genomic windonws. Black points indicate observed pairwise LD values. The blue curve shows the expected decay of LD in the data estimated by nonlinear regression.

Figure S6: Heatmaps showing patterns of LD (r^2) along all chromosomes separately, with darker areas corresponding to genomic regions characterized by elevated values of LD, as shown in the colour key. White lines refers to SNPs that were not polymorphic in a given species, which resulted in a NA value when used to calculated LD measures.

Table S1: Sampling locations of specimens used in these analyses. For each location we provide also species identity and the number of samples that were retained after quality control and filtering.

Table S2: Table showing the priors for all estimated parameters separately for each demographic model.

Table S3: Relative maximum ln(likelihoods), number of estimated parameters (K) and AIC values for the five alternative demographic models. Maximum ln(likelihood) values are given as the best value among the 50 independent runs for each model.

Table S4: Estimated parameter values and their associated 95% confidence intervals (95% CI) from the best supported demographic model of *P. maximus* and *P. jacobaeus*. See the Material and Methods for descriptions of the estimated parameters.

Table S5: Measures of nucleotide diversity (π) calculated for each chromosome separately for *P. maximus* and *P. jacobaeus*.

Table S6: List of all identified fully diagnostic SNP markers together with their locations in the *P. maximus* reference genome as well as the typical allele found in each species.

Table S7: Full report of the GO enrichment analysis based on all SNPs located within highly divergent genomic windows. For each GO term, we also report the term description and the resulting p-value.

Table S8: Full report of the GO enrichment analysis based only on SNPs located within genes located within highly divergent genomic windows. For each GO term, we also report the term description and the resulting p-value.

Table S9: Full annotation report for all SNPs located within highly divergent genomic windows. For each SNPs we report the location within the *P. maximus* reference genome, the mutation type as classified by the snpEff analysis ('Type') as well as its predicted effect ('Effect'), the start and stop position of the gene where the SNP is located in or of the closest gene, the annotation ID as described by Zeng et al. (2021) and the corresponding best hits against the NR, Swissprot and KEGG databases, and finally, whether the SNP is located or not within a gene ('Within_gene').
